# Bayesian inference of sample-specific coexpression networks

**DOI:** 10.1101/gr.279117.124

**Published:** 2024-09

**Authors:** Enakshi Saha, Viola Fanfani, Panagiotis Mandros, Marouen Ben Guebila, Jonas Fischer, Katherine H. Shutta, Dawn L. DeMeo, Camila M. Lopes-Ramos, John Quackenbush

**Affiliations:** 1 Department of Biostatistics, Harvard T.H. Chan School of Public Health, Boston, Massachusetts 02115, USA;; 2 Channing Division of Network Medicine, Brigham and Women's Hospital, Boston, Massachusetts 02115, USA;; 3 Department of Medicine, Harvard Medical School, Boston, Massachusetts 02115, USA;; 4 Department of Data Science, Dana-Farber Cancer Institute, Boston, Massachusetts 02215, USA

## Abstract

Gene regulatory networks (GRNs) are effective tools for inferring complex interactions between molecules that regulate biological processes and hence can provide insights into drivers of biological systems. Inferring coexpression networks is a critical element of GRN inference, as the correlation between expression patterns may indicate that genes are coregulated by common factors. However, methods that estimate coexpression networks generally derive an aggregate network representing the mean regulatory properties of the population and so fail to fully capture population heterogeneity. Bayesian optimized networks obtained by assimilating omic data (BONOBO) is a scalable Bayesian model for deriving individual sample-specific coexpression matrices that recognizes variations in molecular interactions across individuals. For each sample, BONOBO assumes a Gaussian distribution on the log-transformed centered gene expression and a conjugate prior distribution on the sample-specific coexpression matrix constructed from all other samples in the data. Combining the sample-specific gene coexpression with the prior distribution, BONOBO yields a closed-form solution for the posterior distribution of the sample-specific coexpression matrices, thus allowing the analysis of large data sets. We demonstrate BONOBO's utility in several contexts, including analyzing gene regulation in yeast transcription factor knockout studies, the prognostic significance of miRNA–mRNA interaction in human breast cancer subtypes, and sex differences in gene regulation within human thyroid tissue. We find that BONOBO outperforms other methods that have been used for sample-specific coexpression network inference and provides insight into individual differences in the drivers of biological processes.

The majority of human traits and diseases are driven not by individual genes, but by networks of genes and proteins interacting with each other. Understanding how genes interact and cooperate under different conditions is a central challenge in deciphering the complexities of cellular processes and their dysregulation in various diseases. Differential expression analysis with conventional tools such as “limma” or “voom” (Ritchie et al. 2015) has allowed us to investigate changes in gene expression attributable to diseases while simultaneously adjusting for the effects of covariates, including age and sex. However, differences in transcription levels alone often fail to explain biological differences between the cohorts being compared (Glass et al. 2013).

Coexpression networks, which represent the coordinated expression patterns of genes across diverse biological samples, can provide insights into processes that are simultaneously activated in different biological states (Becker et al. 2023). However, most methods for constructing coexpression networks estimate an aggregate network for the entire population (Langfelder and Horvath 2008; Dawson et al. 2012; Lemoine et al. 2021), thus failing to capture the heterogeneous, context-specific gene interactions present within individual samples.

Methods to infer sample-specific networks have been proposed to address some of these limitations. This includes the single Pearson correlation coefficient (SPCC) (Yu et al. 2015; Zhang et al. 2015), which estimates the significance of sample-specific correlations using a *Z*-test; linear interpolation to obtain network estimates for single samples (LIONESS) (Kuijjer et al. 2019), which was developed to infer sample-specific gene regulatory networks (GRNs) but has also been applied to the Pearson correlation coefficient; and the sample-specific-weighted correlation network (SWEET) (Chen et al. 2023), which modifies the LIONESS equation to account for size imbalances in data set subpopulations.

Although useful, these methods can produce coexpression matrices that are not positive-semidefinite or yield coexpression values outside the defined range for correlation measures (e.g., [−1, 1] for Pearson correlation coefficient). Because correlation matrices are positive-semidefinite by definition, this non-positive-semidefiniteness may pose challenges in downstream analyses. For example, if an estimated coexpression matrix is not positive-semidefinite, certain linear projections of gene expression, including several principal components (PCs), might have negative variance estimates. In more extreme situations, the variance of expression values of certain genes might be negative for some individuals, thus making uncertainty quantification impossible. Alternatively, other methods designed for personalized characterization of diseases through sample-specific networks (Liu et al. 2016) and cancer-specific or group-specific networks (Lee et al. 2020) represent differential networks with respect to an external reference population and, hence, are susceptible to varying inference depending on the reference used.

We developed Bayesian optimized networks obtained by assimilating omic data (BONOBO), an empirical Bayesian model that derives individual sample-specific coexpression networks (Fig. 1), facilitating the discovery of gene pairs differentially coregulated between different conditions and phenotypes while eliminating the effects of confounders. BONOBO derives positive-semidefinite coexpression networks from input data alone, without using external reference data sets. This distinctive feature enables BONOBO to capture correlation structures that remain consistent and comparable across diverse data sets and multiple batches, providing a robust tool for correlation network analysis and meaningful correlation networks for input to GRN inference tools. BONOBO derives a posterior probability distribution for individual correlation matrices, allowing us to test the hypothesis of whether any pairs of genes have a nonzero correlation within an individual sample in the data. Based on the results of this hypothesis testing, one can infer individual sample-specific sparse coexpression networks by pruning out nonsignificant edges. This is particularly important for interpretability as empirical data suggest that biological gene networks are sparsely connected (Leclerc 2008).

**Figure 1.**
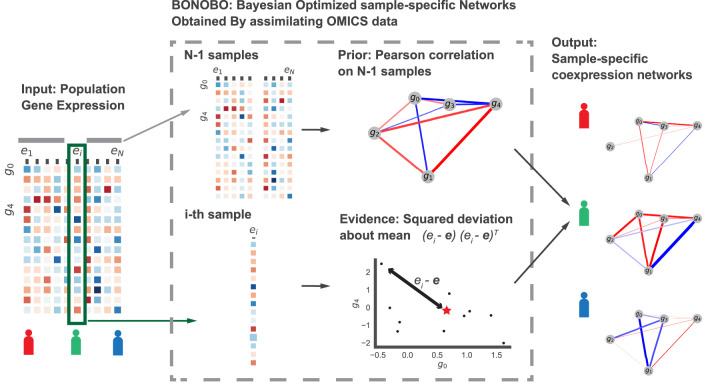
Schematic diagram of BONOBO. BONOBO requires a gene expression matrix as input, from which we would like to extract sample-specific correlation networks. Then, for each of the samples, BONOBO infers the network by using both the Pearson correlation matrix computed on *N* − 1 samples and the sample-specific squared-deviation about the mean. BONOBO outputs *N* coexpression networks, one for each sample, and the associated *P*-values for each of the gene–gene estimated edges.

One of the key strengths of BONOBO lies in its ability to capture the inherent heterogeneity in coexpression patterns among individuals within a population that can be attributed to a range of biological and environmental elements. For example, when comparing aggregate coexpression networks between conditions, such as distinguishing between health and disease or samples from male and female, results are frequently confounded by the study population's heterogeneity stemming from nuisance parameters, such as batch effects or confounding clinical covariates that can include sex or age. BONOBO's individual sample-specific approach explicitly models this heterogeneity, enabling a deeper understanding of the gene networks underlying distinct biological states. In addition, individual sample-specific coexpression networks derived by BONOBO can be used to infer sample-specific GRNs by using the BONOBO networks as an input to methods such as PANDA (Glass et al. 2013), OTTER (Weighill et al. 2021), and EGRET (Weighill et al. 2022).

We demonstrated the advantages of BONOBO using several simulated and real data sets. First, we used simulated data to compare BONOBO's ability to recover “known” sample-specific coexpression matrices with that of other methods for inferring sample-specific networks. We then applied BONOBO to gene expression data from a yeast perturbation experiment and showed that it captures global properties more consistently than other methods and can distinguish the sample-specific effects of single transcription factor knockouts (KOs). Next, we examined the interaction between miRNA and mRNA expression in various human breast cancer subtypes using individual-specific coexpression networks derived by BONOBO and found that the correlation patterns between miRNA expression and immune pathways have prognostic significance in Luminal A and Luminal B breast cancer subtypes. In a final application, we analyzed sex differences in gene regulation using RNA-sequencing data from healthy human thyroid tissue. Using BONOBO networks as inputs to PANDA, we inferred individual-specific GRNs and compared these between males and females, identifying regulatory differences in immune response, cell proliferation, and metabolic processes, thereby providing a possible mechanism for sex bias in incidence rates of various thyroid conditions such as hypothyroidism and Hashimoto's disease (Pizzato et al. 2022).

BONOBO is available as open-source code in Python through the Network Zoo package (netZooPy v0.10.0; https://netzoo.github.io) (Ben Guebila et al. 2023).

## Methods

### BONOBO

Let $x_{1},x_{2},\ldots ,x_{N}∼R^{g}$ denote the *log-transformed* bulk gene expression values of *g* genes for *N* samples. Let us assume that for every sample *i* ∈ {1, 2, …, *N*}, the centered log expression vector $x_{i}-\bar{x}$ follows a multivariate normal distribution with mean zero and an unknown sample-specific covariance matrix *V*_*i*_,

(1) $$x_{i}-\bar{x}∼N_{g}(0_{g},V_{i}),$$

where $0_{g}\in R^{g}$ denotes a vector of all zeros, and $\bar{x}=\frac{1}{n}\sum_{i=1}^{n}x_{i}$ denotes the mean expression across all samples. Our objective is to estimate *V*_*i*_, the sample-specific covariance matrix of gene expression for the *i*th sample, ∀i.

It is worth noting that although microarray expression data are often assumed to follow a Gaussian distribution, this assumption is not valid for the expression count data from RNA-sequencing experiments. However, the *log-transformed* expression data are approximately Gaussian and continuous, so in BONOBO, we impose a multivariate Gaussian assumption on the *log-transformed* data (for a detailed discussion on the validity of this assumption, see Supplemental Material S1.8).

The second assumption we make is that for every sample *i* ∈ {1, 2, …, *N*}, the sample-specific covariance matrix *V*_*i*_ follows an inverse-Wishart prior distribution, given all other samples j∈{1,…,N}∖{i},

(2) $$V_{i}∼InvWishart((\nu_{i}-g-1)S_{i},\nu_{i}),$$

where ν_*i*_ ≥ *g* + 1 denotes the degrees of freedom, and *S*_*i*_ denotes the sample covariance matrix computed from *N* − 1 samples excluding the *i*th sample. Under this assumption, the prior mean of the covariance matrix for the *i*th sample is E[*V*_*i*_] = *S*_*i*_. In other words, we assume that the correlation between any pair of genes for each individual is centered around the correlation between the same pair of genes across the entire population. How much the individual-specific correlation differs from the population-level correlation for every individual depends on the individual-specific degrees of freedom parameter ν_*i*_. In the following section, we describe a data-driven approach to calibrate this parameter so it reflects how similar a particular sample is to the rest of the population, thereby allowing the individual-specific correlation estimates to deviate from the population-level correlation values accordingly.

The inverse-Wishart distribution is a conjugate prior for the covariance matrix of a multivariate normal distribution. Therefore, under the above prior specification, the posterior distribution of the sample-specific covariance matrix *V*_*i*_ also turns out to be an inverse-Wishart distribution, as described by the following theorem.

Theorem 1. Under Assumptions 1 and 2, the posterior distribution of *V*_*i*_ is

(3) $$V_{i}|\{x_{1},\ldots ,x_{N}\}∼InvWishart((\nu_{i}-g)\Sigma_{i},\nu_{i}+1),$$

where $\Sigma_{i}=\frac{(x_{i}-\bar{x}){\left(x_{i}-\bar{x}\right)}^{T}+(\nu_{i}-g-1)S_{i}}{\nu_{i}-g}$ denotes the posterior mean of *V*_*i*_.

The proof of the above theorem is given in the Supplemental Material S1.1.1.

From Equation 3, we observe that the posterior mean of *V*_*i*_, the covariance matrix of the *i*th sample, is a linear combination of the prior mean *S*_*i*_, which summarizes information from all other samples excluding the *i*th sample, and a sample-specific component $(x_{i}-\bar{x}){\left(x_{i}-\bar{x}\right)}^{T}$, which summarizes the association between pairwise genes within the *i*th sample alone:

(4) $$\Sigma_{i}=\delta_{i}(x_{i}-\bar{x}){\left(x_{i}-\bar{x}\right)}^{T}+(1-\delta_{i})S_{i},$$

where $\delta_{i}=\frac{1}{\nu_{i}-g}$. Because ν_*i*_ − *g* ≥ 1, we have 0 < δ_*i*_ ≤ 1, which represents the relative contributions of the sample-specific information and the prior information while estimating the posterior mean of *V*_*i*_.

As sample size *n* increases, the strong law of large numbers implies $S_{i}\overset{a.s.}{\to }\Sigma$, where Σ denotes the population covariance matrix. Thus, the hyperparameter δ_*i*_ quantifies the contribution of the sample-specific information in the posterior mean, whereas the complement 1 − δ_*i*_ quantifies the contribution of the population covariance matrix Σ. For homogeneous populations, we recommend using a smaller value of δ_*i*_ or, equivalently, a larger value of 1 − δ_*i*_, as this would increase the contribution of the population covariance Σ and give robust estimates of the sample-specific covariance *V*_*i*_. On the other hand, if the *i*th sample is an outlier with respect to the rest of the population, we recommend using a large value of δ_*i*_, thereby decreasing estimation bias. Alternatively, we can set δ_*i*_ = δ, ∀i to some arbitrary value between (0, 1). In the following section, we describe a computationally inexpensive data-driven empirical procedure for calibrating δ_*i*_ for every sample.

### Fixing prior degrees of freedom

The hyperparameter $\delta_{i}=\frac{1}{\nu_{i}-g}$ is a one-to-one function of the prior degrees of freedom ν_*i*_. Hence, in order to calibrate δ_*i*_, it suffices to estimate ν_*i*_ for every sample *i* in the data. The following lemma provides a data-driven approach for calibrating ν_*i*_.

Lemma 1. Under Assumption 2, prior variance of the *k*th diagonal entry of *V*_*i*_ (denoted by $v_{i}^{\left(kk\right)}$) would be

(5) $$Var(v_{i}^{\left(kk\right)})=\frac{2{\left(s_{i}^{\left(kk\right)}\right)}^{2}}{\nu_{i}-g-3},$$

where ${\left(s_{i}^{kk}\right)}^{2}$ denotes the *k*th diagonal entry of *S*_*i*_.

The above lemma is a direct consequence of the properties of the inverse-Wishart distribution (Zhang 2021).

From Equation 5, summing over *k* = 1, …, *g* (over all genes) we get

(6) $$\overset{g}{\underset{k=1}{\sum }}Var(v_{i}^{\left(kk\right)})=\frac{2\sum_{k=1}^{g}{\left(s_{i}^{\left(kk\right)}\right)}^{2}}{\nu_{i}-g-3}.$$

Simplifying the above equation gives us,

(7) $$\nu_{i}=g+3+\frac{2\sum_{k=1}^{g}{\left(s_{i}^{\left(kk\right)}\right)}^{2}}{\sum_{k=1}^{g}Var(v_{i}^{\left(kk\right)})}.$$

For every sample *i*, the right side of Equation 7 is known except for $Var(v_{i}^{\left(kk\right)})$ for *k* = 1, …, *g*. We can approximate this value from the data as follows:

1. Get *N* estimates of the variance of the *k*th gene by leaving out one sample at a time: $\left\{\eta_{1}^{k},\eta_{2}^{k},\ldots ,\eta_{N}^{k}\right\}$, where $\eta_{j}^{k}$ denotes the variance of *k*th coordinates of $\{x_{1},\ldots ,x_{N}\}\setminus x_{j}$.
2. Estimate $\eta^{\left(k\right)}=\frac{1}{N}\sum_{\;j=1}^{N}{\left(\eta_{j}^{k}-\frac{1}{N}\sum_{l=1}^{N}\eta_{l}^{k}\right)}^{2}$, the variance of $\left\{\eta_{1}^{k},\eta_{2}^{k},\ldots ,\eta_{N}^{k}\right\}$.

Replacing $Var(v_{i}^{\left(kk\right)})=\eta^{\left(k\right)}$ on the right-hand side of Equation 7 gives us a data-driven estimate of the prior degrees of freedom ν_*i*_. Thus, the estimate of the hyperparameter δ_*i*_ becomes

(8) $$\delta_{i}=\frac{1}{\nu_{i}-g}=1/\left[3+\frac{2\sum_{k=1}^{g}{\left(s_{i}^{\left(kk\right)}\right)}^{2}}{\sum_{k=1}^{g}\eta^{\left(k\right)}}\right],\;\forall i\in \{1,\ldots ,N\}.$$

In section S1.2.6 of the Supplemental Material, we use simulation experiments to illustrate that this data-driven empirical approach of calibrating δ_*i*_ separately for each individual sample *i* delivers better or comparable performance than assigning a fixed value of $\delta_{i}=\delta ,\forall i$ for all individuals. This empirical data-driven approach for calibrating the degrees of freedom parameters for the individual-specific coexpression matrices allows BONOBO to more accurately model how individual sample coexpression deviates from the population coexpression, thereby more effectively capturing heterogeneity in gene–gene interaction patterns across the population. For a mathematical explanation of why this data-driven approach is effective, see Supplemental Material S1.2.6.

### Hypothesis testing

For every sample *i*, we can derive a 100(1 − α)% posterior credible region for the correlation between any pair of genes as follows: First, we compute the posterior variance of the covariance between any pairs of genes using the following lemma, which is a direct consequence of the properties of inverse-Wishart distribution (Zhang 2021). For simplicity, we remove the sample index *i*.

Lemma 2. Let *v*_*jk*_ denote the covariance between the *j*th and the *k*th gene. Under Assumptions 1 and 2, the posterior variance of *v*_*jk*_ is

(9) $$Var(v_{\;jk})=\frac{(\nu -g+1)s_{\;jk}^{2}+(\nu -g-1)s_{\;jj}s_{kk}}{\left(\nu -g)(\nu -g-3\right)},$$

where *s*_*kk*_ denotes the *k*th diagonal entry of the prior mean *S*, and *s*_*jk*_ denotes the (*j*, *k*)th off-diagonal entry (corresponding to the *j*th row and the *k*th column) of *S*.

Using the above lemma we can compute an approximate 100(1 − α)% posterior credible region for *v*_*jk*_ as (σ_*jk*_ − ψ_*jk*_*z*_(1−α/2)_, σ_*jk*_ + ψ_*jk*_*z*_(1−α/2)_), where $\psi_{\;jk}=\sqrt{Var(v_{\;jk})}$, σ_*jk*_ is the posterior mean of *v*_*jk*_ and *z*_(1−α/2)_ denotes the (1 − α/2)th quantile of the standard normal distribution.

We use the central limit theorem (CLT) to derive the posterior credible regions. Even though the posterior distribution of each individual-specific coexpression matrix is known, the closed-form expressions of the quantiles of inverse-Wishart distribution are theoretically intractable, and unlike many other distributions, such as Gaussian, no software implementation is available for inverse-Wishart quantiles. To construct a posterior credible region for each individual, we need to simulate a large number of samples from the corresponding inverse-Wishart posterior, which is computationally expensive. Moreover, this credible region constructed from simulated observations would give us the joint credible region for the correlation between all pairs of genes and not the marginal credible region for each pair of genes, which we are interested in estimating as it is biologically more interpretable. Therefore, we use the CLT to derive a computationally inexpensive credible region.

In low dimensional settings, Bayesian credible regions can be used as frequentist confidence intervals, as justified in section 10.2 of the work by Van der Vaart (2000). Thus, we can reject the null hypothesis *H*_0_:*v*_*jk*_ = 0, in favor of the alternative hypothesis *H*_1_:*v*_*jk*_ ≠ 0 at significance level α, provided 2(1 − Φ(σ_*jk*_/ψ_*jk*_)) ≤ α, where Φ denotes the cumulative distribution function of the standard normal distribution. In practice, BONOBO derives a dense (complete) network with edges between every pair of genes, where edge weights correspond to σ_*jk*_, the posterior mean of the covariance between genes *j* and *k*. We can generate a sparse covariance network by simply pruning out edges for which the 100(1 − α)% posterior credible regions contain zero for a suitable value of 0 < α < 1.

### Computing coexpression from covariance

For every sample *i*, BONOBO first computes a sample-specific covariance matrix *V*_*i*_ following the abovementioned procedure. These sample-specific covariance matrices can be subsequently converted to the corresponding sample-specific correlation (generally coexpression) matrices as follows. For each pair of genes, we can compute the sample-specific coexpression simply by dividing the covariance between the two genes by the product of the standard deviations of these same two genes. Formally speaking, for sample *i*, the correlation between genes *j* and *k* would be $\frac{v_{\;jk}^{\left(i\right)}}{\sqrt{v_{\;jj}^{\left(i\right)}v_{kk}^{\left(i\right)}}}$, where $v_{\;jk}^{\left(i\right)}$ denotes the (*j*, *k*)th entry of the covariance matrix *V*_*i*_.

## Results

### Simulated data and comparison with other methods

Although we recognize that simulated GRNs may not capture the full complexity of the gene expression and the effects of regulation, simulation is an important tool as it provides a measure of ground truth against which various methods can be rigorously benchmarked and compared. We performed five simulation experiments and compared BONOBO with three other methods for computing sample-specific coexpression: LIONESS (here used only in the LIONESS::Pearson configuration), SPCC, and SWEET. We repeated each of the simulation experiments 100 times and compared BONOBO with LIONESS::Pearson, SPCC, and SWEET based on the mean sum of squared errors (the squared Frobenius norm of the difference between the true correlation matrix and the estimated correlation matrix) across these 100 iterations.

**Figure 2.**
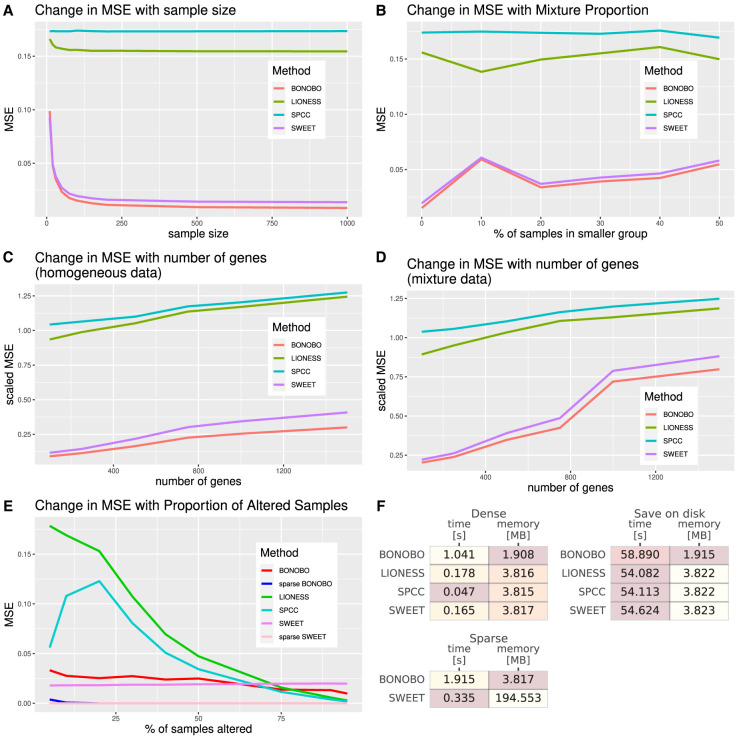
Comparisons between BONOBO and other methods for the estimation of sample-specific coexpression on simulated data. Performance of BONOBO, LIONESS::Pearson, SPCC, and SWEET on simulated data. (*A*) Simulated data from homogeneous population: change in MSE with respect to sample size. (*B*) Simulated data from a mixture of two populations: change in MSE with respect to the percentage of samples in the smaller population. (*C*) Simulated data from a homogeneous population: change in scaled MSE (MSE divided by the variance) with respect to the number of genes. (*D*) Simulated data from a mixture of two populations, in which 20% of samples come from one population and the remaining 80% of samples come from another population with distinct mean and covariance of gene expression: change in scaled MSE with respect to the number of genes. (*E*) Simulated data in which some samples lost expression of 1% of genes: a change in MSE with respect to the proportion of altered samples. BONOBO outperforms all three competing methods by providing lower MSE across varying sample sizes, varying mixture proportions of subpopulations, and varying the number of genes. (*F*) Computational resources required to run each method: We report average time and memory consumption measured over 10 data sets, with 500 genes and 100 samples; darker colors correspond to better performance/lower resources. We first compare resources to infer “dense” networks, the standard implementation of each method, and those needed to infer “sparse” BONOBO and SWEET. Then, we compare the resources required to infer and save to disk each network (save on disk), which is a more realistic scenario when dealing with larger data sets. BONOBO consistently performs better in terms of required memory, although it is slower than the others. However, we can see that when networks are saved on disk, which is a time-consuming task, BONOBO's disadvantage is greatly reduced.

First, we examined how the performance of BONOBO compares with the competing methods for varying sample sizes, based on 100-dimensional gene expression data generated from a homogeneous population (Supplemental Material S1.2.1). We found that as sample size increases from 10 through 1000, the mean squared error (MSE) declines for BONOBO, LIONESS::Pearson, and SWEET and remains unchanged for SPCC (Fig. 2A). For any sample size, BONOBO outperforms all competing methods, although the performance improvement over SWEET is much smaller compared with the performance gains over LIONESS::Pearson and SPCC. However, upon closer inspection (Supplemental Fig. S1, top left) one can see that not only does BONOBO provide a steady decrease in MSE over SWEET across all sample sizes, but as sample size increases, BONOBO exhibits increasingly better performance compared with SWEET by providing up to a 40% decrease in MSE for sample size = 1000.

Next, we simulated samples from a mixture of two different homogeneous populations (Supplemental Material S1.2.2) and demonstrated that the performance of BONOBO, along with SWEET and LIONESS::Pearson, declines as the proportion of samples from the smaller subpopulation increases, meaning that the overall population becomes more diverse (Fig. 2B). For any given mixture proportions of subpopulations, the MSEs for BONOBO were much smaller than those for LIONESS::Pearson and SPCC, whereas SWEET performed slightly worse than BONOBO (Supplemental Fig. S1, top right).

Next, we compared the performance of BONOBO to the other methods by keeping sample sizes fixed at *n* = 100 and varying the number of genes from 100 to 1500. We examined two different situations: the first using samples drawn from a homogeneous population (Supplemental Material S1.2.3) and the second in which samples are drawn with a 20:80 mix from two homogeneous subpopulations (Supplemental Material S1.2.3) One can see that the performance of all four methods declines as the number of genes increases (Fig. 2C,D). However, for any fixed number of genes, BONOBO has a smaller MSE compared with the other methods. Further, as the number of genes is increased, the difference between the MSE of BONOBO and the MSE of the closest competitor, SWEET, grows (Supplemental Fig. S1, bottom right) such that BONOBO's performance as we approach genome-scale analyses becomes substantial.

In the next example, we simulated samples from a mixture of two populations, in which one population lost expression for 1% of genes. The sparse version of BONOBO identified this loss of expression (Supplemental Material S1.2.5) with better accuracy compared with BONOBO without sparsity, as well as other nonsparse competitors including SWEET, LIONESS::Pearson, and SPCC, whereas both sparse SWEET and sparse BONOBO had comparable performance (Fig. 2E).

Additionally, we used two simulation examples to illustrate (Supplemental Material S1.2.6) that the data-driven approach of calibrating hyperparameters δ_*i*_ for each sample *i* described in the Methods subsection entitled “Fixing prior degrees of freedom,” provides a better estimation accuracy compared with the performance obtained by choosing a fixed value of $\delta_{i}=\delta ,\forall i$ for all samples.

We also compared the computational performance of the four methods. We generated 100 simulated data sets with 500 genes and 100 samples each; applied BONOBO, LIONESS::Pearson, SPCC, and SWEET to each data set (see Supplemental Material S1.3); and measured how much time and memory each method required on average (Fig. 2F). We found BONOBO to be more memory efficient than the other methods, as it only needs to store one gene-by-gene matrix in memory. For the sparse case, BONOBO must store another gene-by-gene *P*-value matrix, effectively doubling the memory requirement. SWEET uses an even more inefficient strategy, simultaneously keeping all networks in memory. When considering the speed of execution, BONOBO was slower than the other methods, but this is somewhat mitigated by the fact that saving each network on disk, which is necessary when dealing with real data, adds a considerable time overhead to the computation (an additional 50 sec), hence reducing the speed advantage of SWEET, LIONESS::Pearson, and SPCC. For completeness, we repeated the same analyses for varying numbers of genes and samples (Supplemental Figs. S3–S5), confirming the trend.

The simulated data sets we analyzed resemble many scenarios that are encountered in the analysis of biological data sets, including different population sizes, gene KOs and silencing, and mixtures of subpopulations. In all these conditions, BONOBO performs as well as, or better than, LIONESS::Pearson, SPCC, and SWEET. Further, although the relative performance advantage of BONOBO in some conditions is minor and its run time is greater than the other methods, BONOBO is more memory efficient, making it scalable for use with genome-wide correlation networks in situations in which memory limitations would prevent use of the other methods.

### BONOBO recovers sample-specific network structure in yeast data sets

Having assessed the performance of BONOBO relative to other methods for inferring single-sample correlation networks using simulated data, we then explored its performance in analyzing correlation networks in real-world data. *Saccharomyces cerevisiae* (brewer's yeast) has been extensively used to explore gene function and to explore various network models (Reimand et al. 2010; Hahn and Young 2011; Costanzo et al. 2016, 2021; Teixeira et al. 2018; Hackett et al. 2020), and so, we chose expression data from two experiments to test how well BONOBO could find biologically relevant changes in gene expression correlation patterns. As a baseline test, we used BONOBO to estimate correlation networks using microarray expression data from synchronized yeast cells at 48 time points (Pramila et al. 2006) and found that BONOBO was able to accurately identify both cyclic fluctuations in cell-cycle-stage transition pathways and to identify biologically meaningful changes in functionally relevant groups of genes (see Supplemental Material S1.4).

We then turned our attention to a yeast gene perturbation experiment in which eleven transcription factors were knocked out (11 KO strains), and each was grown in 12 different growth media (Jackson et al. 2020). The 11 TF KO targets include known regulators of the nitrogen catabolite repression (NCR) pathway, the general amino acid control (GAAC) pathway, the Ssy1-Ptr3-Ssy5-sensing (SPS) pathway, and the retrograde pathway. The 12 different growth media represent a number of nitrogen and carbon sources (Supplemental Material S1.5). Because the original data came from a single-cell RNA-seq analysis, we created pseudobulked expression data for the 132 KO/media combinations. Jackson et al. (2020) reported that the transcriptional effects of changing growth medium (and carbon or nitrogen source) had a greater effect than did the individual TF KOs. This is expected because the nutrients included or excluded from each growth medium will specifically activate genome-wide metabolic pathways, although knocking out one TF is likely to produce localized changes among that TF's gene targets.

Consistent with expectations, we found that BONOBO's networks were far more similar for yeast in the same growth medium irrespective of TF KO than for strains with the same KO irrespective of growth media (see Supplemental Fig. S9). Going beyond this qualitative observation, we can use this hypothesis; that is, same-media networks should be more similar to each other than the other networks, as ground truth to assess the relative performance of BONOBO and other single-sample correlation network estimation methods. To do this, we used multiple quantitative methods, including clustering, correlation, and distance metrics, to assess the global similarities between sample-specific networks (Supplemental Material S1.5.1).

**Figure 3.**
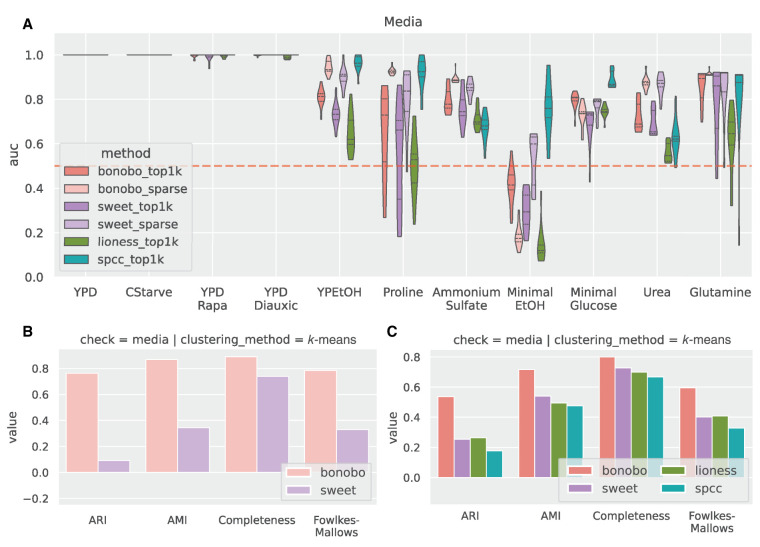
Comparison between methods for detecting media perturbations in yeast experiments. We used only the edges returned by the sparsified versions of SWEET and BONOBO and the 1000 strongest edges, on average, for each method (top1k labels). (*A*) Network similarity between perturbed yeast samples. For each sample, we computed Pearson correlation coefficients with all the other samples, which is always positive here, and estimated the ROC curve using a binary label that is one for the samples grown in the same media. For each comparison, we computed the AUROC (*y*-axis), grouping the samples by the growth medium (*x*-axis), and we compared the results between different methods. *auc* = 1 means that samples in the same media have the highest correlation values compared with samples in different media, *auc* = 0.5 (red dashed line) is the “random” performance, which means that samples with the same label are not more similar to each other than the rest. (*B*) Clustering performance on sparse BONOBO and SWEET. For all networks, we used *k*-means clustering and evaluated how well the resulting clusters captured growth media similarity using four different metrics: adjusted rand score (ARI), adjusted mutual info score (AMI), completeness, and Fowlkes–Mallows score. Greater values indicate that the clusters group samples grown in the same media. (*C*) Clustering performance on top1k networks using the same protocol as in *B*. Sparse networks, both for SWEET and BONOBO, outperform the naive “top1k” networks, with BONOBO outperforming SWEET. Although for some growth media, SPCC has a greater *auc* than BONOBO, BONOBO outperforms SPCC in the clustering test.

As in the simulations, we computed both sparse and dense networks, then computed pairwise correlations and distances between all the networks derived for each sample (Fig. 3A), and used a number of clustering methods (*k*-means, spectral, and agglomeration clustering) to determine how well correlation matrices inferred using each method reflected the growth media effect (Fig. 3B,C). We found that BONOBO more consistently detected these growth media similarity patterns than did the other methods and that the sparsification statistical framework we described provides more manageable networks while maintaining strong performance (Fig. 3; Supplemental Figs. S11, S12).

None of the single-sample correlation network methods were able to pick out the weak similarities caused by TF KOs as they were masked by the signal from the growth media (Supplemental Figs. S11–S13). However, we reasoned that if we controlled the analysis for the effect of the growth media, we could discern the effects of TF KO. Looking at each media individually, we tested whether genetically perturbed samples showed stronger perturbations in the edges connected to the knocked-out TF. For each growth medium and each TF, we selected only the edges with the highest weights and tested whether these edges in the wild-type were significantly different compared to the perturbed samples using Kolmogorov–Smirnov tests. We summarized these results by computing the F1 score; where for each TF KO, we computed the true-positive (TP) rate as the proportion of edges connected to that specific TF that are significantly different between wild-type and the perturbed samples with that specific TF knocked out (see Supplemental Material S1.5; Supplemental Fig. S16). We found that although all methods were able to detect KO effects with generally comparable performance, BONOBO networks were consistently better at detecting changes resulting from the KO of the TFs *GCN4*, *GAT1*, *GLN3*, and *RTG3*.

It is worth noting that the BONOBO model explicitly assumes that log-transformed gene expression values have a Gaussian distribution. However, after using the Kolmogorov–Smirnov test, we were unable to reject the Gaussian distribution null hypothesis for only 66.61% of genes (Supplemental Fig. S25, right panel). Nevertheless, BONOBO performed at least comparably to, and sometimes even better than, the other methods in all the tests we performed using yeast data.

Finally, we wanted to understand what BONOBO's sample-specific coexpression networks can reveal about how a TF KO affects the overall network (Supplemental Fig. S17). This is a challenging task because we have shown that the effect of changing growth medium is greater in most instances than knocking out a TF. The one possible exception in this data set is the deletion of *GCN4*, which produced easily detectable changes in the correlation patterns of the genes that are most strongly correlated with *GCN4* across samples (Supplemental Fig. S18). We selected the 100 genes whose correlation edge weights differed the most between the *GCN4* KO and the rest of the data set. As expected, genes that were most perturbed by *GCN4* deletion, which targets the GAAC pathway, belong to many pathways related to autophagy, such as exosome, phagosome, and autophagy–yeast (Supplemental Fig. S18). Among the most significantly perturbed genes were known GCN4 targets (Wong et al. 2023), including *VCX1*, *MNN10*, *ACT1*, *CPA1* (Uluisik et al. 2011; Coey and Clark 2022), and *CCW12* (Rawal et al. 2018), demonstrating that perturbation-specific coexpression networks estimated using BONOBO can be used to detect key regulatory targets of the TF.

### miRNA–gene interaction in breast cancer subtypes

We also wanted to explore the application of BONOBO to multiomic data sets while accounting for sample-specific clinical and molecular confounders. MicroRNAs (miRNAs) play an important regulatory role through RNA silencing, resulting in the downregulation of their target genes; changes in miRNA expression levels and their downstream effects have been shown to play a role in cancer development and are predictive of outcomes in some cancers (Oliveto et al. 2017). We calculated coexpression networks using paired mRNA and miRNA expression data from 101 breast cancer samples representing the five canonical molecular subtypes (obtained from the NCBI Gene Expression Omnibus [GEO; https://www.ncbi.nlm.nih.gov/geo/] accession number GSE19783) (for preprocessing steps, see Supplemental Material S1.6.1; Enerly et al. 2011; Aure et al. 2013; Haakensen et al. 2016b). We then used BONOBO to infer sample-specific coexpression networks to explore how correlation between miRNA and gene expression vary between breast cancer subtypes (normal-like, Luminal A, Luminal B, ERBB2/HER2-positive, and basal) and whether patterns of coexpression can be used to predict survival outcomes.

BONOBO produced 101 sample-specific coexpression networks, each of which includes correlations between pairs of genes, between pairs of miRNAs, and between each gene and each miRNA. We compared the miRNA–gene edges between subtypes. For each network, we ranked the genes by their “miRNA-specific degree” and performed preranked gene set enrichment analysis (Supplemental Material S1.6.2). A gene's “miRNA-specific degree” is calculated by summing all coexpression values between that gene and each of the miRNAs. For instance, if for a particular sample, $\left\{\rho_{g_{j}m_{1}},\rho_{g_{j}m_{2}},\ldots ,\rho_{g_{j}m_{K}}\right\}$, represents the correlations between gene *g*_*j*_ with miRNAs *m*_1_, …, *m*_*K*_, respectively, then the total correlation between gene *g*_*j*_ with miRNAs is computed as $\sum_{k=1}^{K}\rho_{g_{j}m_{k}}$. By ranking genes in this way and performing pathway enrichment analysis, we can identify those biological pathways that are significantly either positively or negatively correlated with miRNA expression.

We found that pathways associated with immune response, including graft-versus-host disease, primary immunodeficiency, cytokine–cytokine receptor interaction, and natural killer cell–mediated cytotoxicity, were significantly negatively correlated (FDR cutoff 0.05) with miRNA expression across all breast cancer subtypes (Supplemental Fig. S20). Pathways associated with cell adhesion and cell proliferation, such as focal adhesion and ECM receptor interaction, were positively correlated with miRNA expression, especially in the basal, normal-like, and Luminal B subtypes, whereas pathways associated with cell adhesion molecules were significantly negatively correlated (FDR cutoff 0.05) with miRNA expression in ERBB2, Luminal A, and normal-like breast cancer. These findings are consistent with previous studies (Enerly et al. 2011) that reported significant associations between the expression levels of several miRNAs and biological pathways involved in cell proliferation, cell adhesion, and immune response.

**Figure 4.**
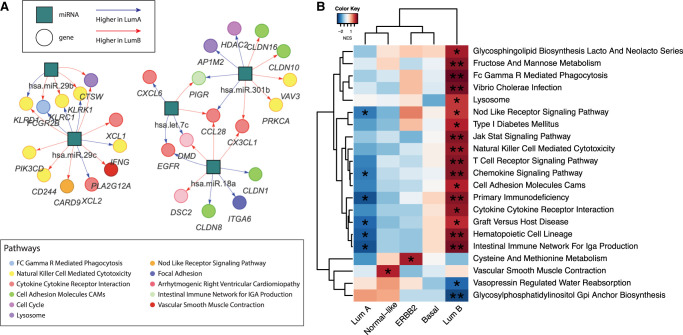
BONOBO networks reveal distinct miRNA–mRNA coexpression patterns in different breast cancer subtypes. (*A*) Pairs of genes and miRNAs that are most differentially coexpressed between Luminal A and Luminal B subtypes: Gene nodes are represented by colored circles, where the colors correspond to the biological pathway associated with that gene; miRNAs are represented by teal squares. Edges are colored red if they have higher weights in Luminal A compared with Luminal B and are colored blue if its miRNA-specific degree is greater in Luminal B than in Luminal A. (*B*) Pathways for which the mean correlation with miRNAs is significantly (at significance level 0.05) associated with survival in a Cox proportional hazard model: Heatmaps are colored by the *t*-statistic of the pathway score in the Cox model. Rows correspond to pathways, and columns represent the breast cancer subtypes. Pathways for which a higher correlation with miRNAs is associated with better survival are colored blue, and pathways for which a higher correlation with miRNAs is associated with worse survival are colored red.

Although the pathways most strongly correlated (positively or negatively) with miRNA expression were consistent between subtypes, when we more closely inspected the individual miRNA–mRNA edges in the networks, we found that there are significant differences between subtypes in the neighborhood of individual genes. For example, when we compared individual correlation edges between genes and miRNAs in Luminal A and Luminal B breast cancer (Fig. 4A), we found several genes that were differentially correlated with miRNA expression between the subtypes. Genes most differentially coexpressed with miRNAs include the proto-oncogene *EGFR*; genes involved in immune response such as *PI3KCD* and *IFNG*; and genes involved in cell–cell adhesion and cell proliferation such as *CLDN1*, *CLDN8*, *CLDN10*, *CLDN16*, etc. These differences between miRNA–gene interactions highlight the distinct molecular landscapes of Luminal A and Luminal B (Haakensen et al. 2016a).

We next investigated if the strength of association between miRNA expression and biological pathways is predictive of survival in each of the subtypes. For every pathway significantly (FDR cutoff 0.05) correlated (positively or negatively) with miRNA expression, we computed a pathway score, defined by the mean of the total correlation between all miRNAs and each gene in that pathway. Then, for each pathway, we fit a Cox proportional hazard model, allowing the coefficients of the Cox model to be subtype-specific. We found that a high correlation between miRNA and immune-related pathways, including the Chemokine signaling pathway, primary immunodeficiency, graft-versus-host disease, hematopoietic cell lineage, and intestinal immune network for IgA production, was associated with better survival among samples from Luminal A but with worse survival among samples from Luminal B subtype (Fig. 4B). Previous studies (Arun et al. 2022) have reported that an increased expression of miRNAs is associated with tumor suppression among Luminal A breast cancers, thus leading to slower tumor growth and improved prognosis. Our analysis indicates that an up-regulation of immune pathways by miRNAs might provide a possible mechanism to explain the tumor-suppressive effect of miRNAs in Luminal A. In Luminal B breast cancer, miRNAs may have a role in regulating immune evasion processes (Gomarasca et al. 2020), resulting in more aggressive tumor growth and poorer survival outcomes.

Previous studies (Zhu et al. 2019) have identified specific immune gene expression patterns that distinguish Luminal A from Luminal B breast cancer and showed that these distinct immune signatures were associated with a differential ratio between *ESR1* and *ESR2*, a higher value of which has been associated with poorer survival outcomes. Our results indicate that subtype-specific regulatory interactions between miRNAs and immune pathways in Luminal A breast cancers might contribute to estrogen receptor–mediated survival outcomes. We also found that the genes linked to cell proliferation pathways exhibit distinct patterns of regulation by miRNAs in Luminal A compared with Luminal B breast cancer. Clinically, this disparity in the regulation of cell proliferation genes might be a crucial factor contributing to poorer prognosis in Luminal B breast cancer patients (Ades et al. 2014).

### Sample-specific GRNs identify sex difference in thyroid

Many thyroid disorders exhibit sex-based differences in incidence, with females being significantly more susceptible to thyroid conditions than age-matched males (Vanderpump 2011). Although sex chromosomes, sex hormones, and the immune system (Shobab et al. 2022) have often been suggested as possible contributors to this sex difference in thyroid tissue, there are few published system-based analyses exploring the molecular mechanisms driving the sex differences.

To fill this gap in our understanding of thyroid diseases, we used data from the Genotype-Tissue Expression (GTEx) Project (for all preprocessing steps, see Supplemental Material S1.7.1; Lonsdale et al. 2013) to calculate individual-specific gene–gene correlation matrices using BONOBO. These coexpression matrices were subsequently combined with TF-motif prior regulatory networks (Supplemental Material S1.7.2) and with TF protein–protein interaction data (Supplemental Material S1.7.1) as input to PANDA (Glass et al. 2013) to estimate 653 sample-specific GRNs (linking TFs to target genes) in healthy research subjects (Supplemental Fig. S21). We performed differential targeting analysis comparing male and female networks (Supplemental Material S1.7.4) and identified several genes with known relevance to thyroid cancers and autoimmune conditions (Supplemental Fig. S23).

For example, the long noncoding RNA *XIST* was more highly targeted in females. Previously, *XIST* has been reported to promote oncogenic activity in papillary thyroid carcinomas (Cai et al. 2023). Among genes highly targeted in males, the tumor-suppressor gene *KDM6A* is known to regulate multiple genes involved in immune response, suggesting its potential influence on the risks of developing various autoimmune conditions (Itoh et al. 2019). Among the other genes more highly targeted in males are *PCM1*, *KMT2C*, and *SOS1*. A mutation in *PCM1* has been associated with papillary thyroid carcinoma (Eng et al. 2023); a mutation in the *KMT2C* gene has been identified as a molecular marker for primary thyroid osteosarcoma (Wang et al. 2022); and overexpression of *SOS1* has been found to promote cell proliferation and cell apoptosis in papillary thyroid carcinoma cells (Pang and Yang 2021).

**Figure 5.**
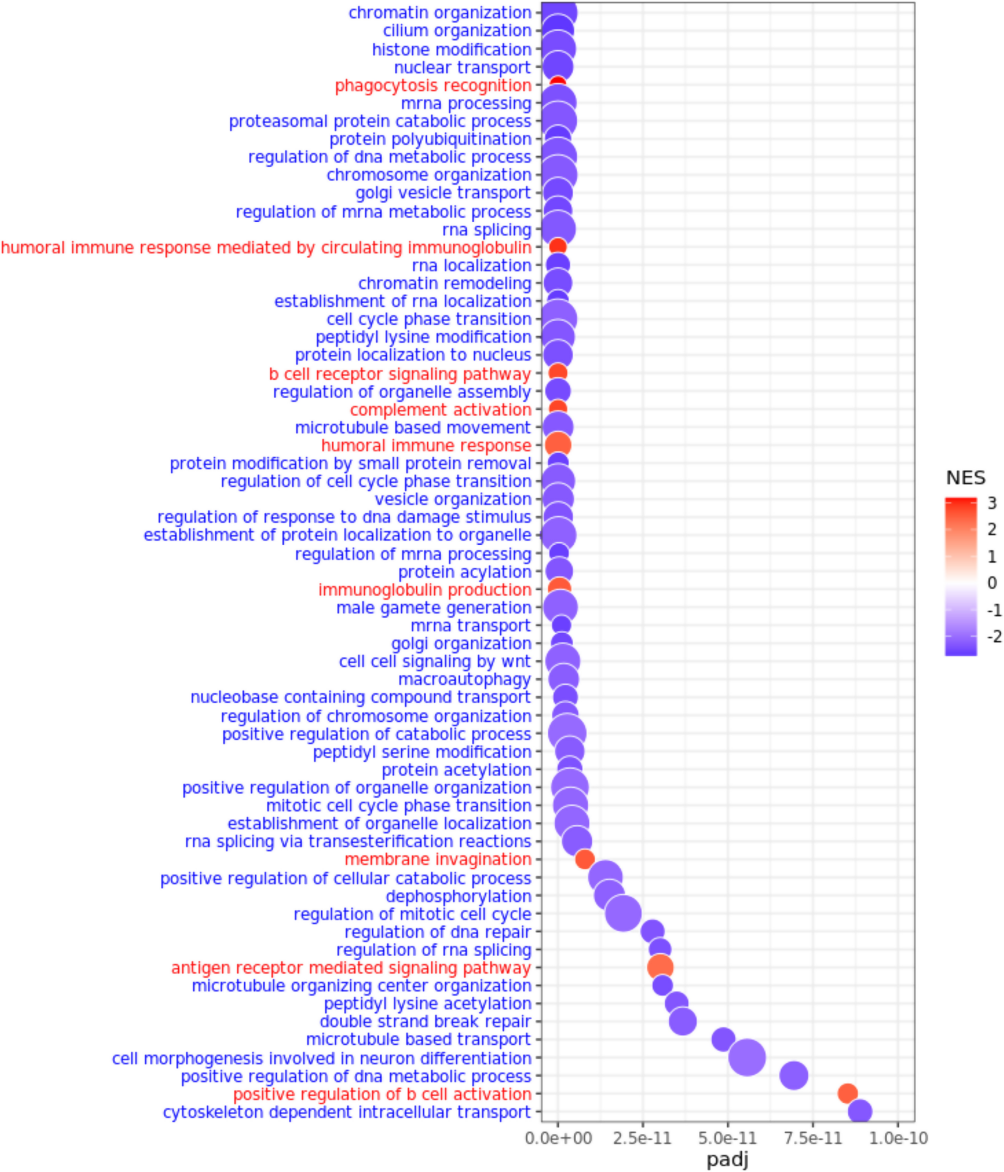
Differential regulation between males and females in healthy thyroid samples. GO biological processes most differentially regulated (at FDR cutoff 1 × 10^−10^) in males and females in GTEx thyroid samples. Pathways are ordered from *top* to *bottom* in ascending order of adjusted *P*-values (padj) such that pathways represented at the *top* are most differentially targeted by transcription factors in males and females. Pathways highly targeted in males are marked in blue, and pathways highly targeted in females are marked in red.

Using genes differentially targeted between the sexes that were ranked by limma *t*-statistics, we performed gene set enrichment analysis using Gene Ontology biological process (GOBP) annotations (see Supplemental Material S1.7.5). Using genes more highly targeted in females, we found enrichment for biological processes associated with immune response, such as humoral immune response, B cell receptor signaling pathway, antigen-receptor-mediated signaling, and positive regulation of the B cell activation pathway (Fig. 5); higher relative targeting of immune pathways in females may contribute to their increased susceptibility to autoimmune thyroid diseases, including Hashimoto's thyroiditis disease, which often leads to hypothyroidism.

For the genes more highly targeted in males, we found enrichment for processes associated with cell cycle, cell signaling, metabolic processes, and DNA repair (Fig. 5); dysregulation of these pathways has been shown to play functional roles in various thyroid conditions, including Graves’ disease and Hashimoto's thyroiditis (Zheng et al. 2022). Although validation experiments are needed to determine the direction of these effects, the differential targeting of these pathways in males suggests that they may have a more robust defense against factors that could disrupt normal thyroid functioning.

To emphasize the advantage of using individual-specific networks as opposed to population-level networks for sex-difference analyses, we performed a gene set enrichment analysis based on sex-specific population-level GRNs constructed separately for males and females using PANDA. Comparing sex-biased biological pathways discovered by individual-specific BONOBO networks and pathways discovered by population-level sex-specific GRNs, we found 12 pathways, all associated with immune response (Supplemental Table S2.2), for which the apparent direction of sex bias was different in the two methods. Examining individual-specific networks, we found that these pathways are highly targeted by TFs in females compared with males, whereas the comparison of sex-specific aggregate networks found the same pathways to be more highly targeted in males.

To understand the reason for this difference for the 12 functional classes, we looked at the average pathway-targeting score (defined as the mean of edge weights connecting TFs to all genes in these 12 pathways) and discovered that males have more outliers with higher targeting scores than females (Supplemental Fig. S24). It is the presence of these outliers that led to the conclusion that these processes are more highly targeted in males, when looking at aggregate networks, even though in reality the targeting scores in females are significantly higher than those in males (*P*-value of Wilcoxon rank-sum test = 0.016).

In fact, greater targeting of immune-related pathways in females has been reported previously in other studies involving individual-specific regulatory networks (Lopes-Ramos et al. 2020; Saha et al. 2024). Biologically, it makes sense that females would have a higher targeting of immune-related pathways because greater targeting could make immune processes in females more susceptible to disruption, giving rise to more frequent immune-related ailments in thyroid tissue such as Hashimoto's disease and other autoimmune conditions.

Overall, individual-specific GRNs derived by combining BONOBO with PANDA identify biological pathways that are differentially regulated by sex and are not discernible from the analysis of population-level sex-specific networks. This sex-biased differential regulation of key genes and pathways might contribute to the differential risk of various thyroid conditions between biological males and females.

## Discussion

Complex human traits and diseases are most often driven by not a single gene but rather by intricate interactions involving multiple genes and the transcription factors that regulate them. Most network inference methods estimate an aggregate network (Langfelder and Horvath 2008; Lemoine et al. 2021) that reflects the average characteristics of the population and ignores the diversity that exists in gene expression and regulation (Saha et al. 2024). Other methods that have been used to infer individual-specific coexpression networks (Kuijjer et al. 2019; Chen et al. 2023) can produce extreme edge weights that are difficult to interpret as correlations. In that sense, BONOBO fills an important gap in methods by estimating true correlation matrices that are positive-semidefinite by definition (in the univariate setting, this translates to expression of individual genes having nonnegative variance). Indeed, for the yeast KO experiment, the SWEET, LIONESS::Pearson, and SPCC networks all have negative eigenvalues (Supplemental Fig. S8), representing negative variances of the PCs of gene expression. In contrast, because BONOBO estimated positive-semidefinite coexpression matrices for every individual, any linear combination of genes, not just PCs, is ensured to have nonnegative variances. Moreover, matrix operations, including matrix inversion and Cholesky decomposition, are interpretable and numerically stable only for positive-semidefinite matrices. Thus, unlike other methods, coexpression matrices inferred by BONOBO can be inverted to compute individual-specific partial correlation networks, which are more effective in discerning direct and indirect interactions between pairs of genes, compared with correlation networks (Shutta et al. 2023). Furthermore, it has been shown that using correctly defined distances on a Riemannian manifold between the covariances from scRNA-seq data improves the discovery of differential gene programs (Ozbay et al. 2023). This approach, which leverages Riemannian geometry of positive-semidefinite matrices, could be extended to detect differences between sample-specific networks derived by BONOBO and not any other existing competing methods. Lastly, because BONOBO is a probabilistic model, one can generate synthetic samples for every individual, which can further be used for sample-specific inference in other contexts.

Constructing individual-specific coexpression networks is challenging as each individual typically has only a single sample. Bayesian statistics enables us to overcome this problem by incorporating prior information derived from other individuals in the same data set. We assume that individual-level covariance matrices come from an inverse-Wishart prior whose mean is equal to the sample covariance matrix computed from all other individuals in the given data set. This assumption is based on the fact that samples in a bulk expression data set typically come from a single tissue and from individuals having similar conditions. Integrating this prior information with the individual-level expression data, BONOBO can estimate the posterior distribution of the covariance matrix for each individual in the data. The mean of the posterior distribution is a weighted average of the deviation of the individual expression from the mean expression and the estimated population covariance from all the other individuals in the data set.

BONOBO is also highly scalable owing to the use of a conjugate prior distribution over individual-specific covariance matrices, which enables us to derive a closed-form expression of the posterior distribution, thus eliminating the need for running computationally expensive Markov chain Monte Carlo. In addition, the posterior distribution of the covariance matrix for each individual is computed separately, without any influence of the posterior distribution of other individuals. These properties, inherent to BONOBO, make the method highly parallelizable and enhance computational efficacy.

BONOBO is based on minimal assumptions and only one tuning parameter that can be efficiently calibrated using a data-driven approach. BONOBO assumes that for every individual in the data, the log-transformed expression values of genes follow multivariate Gaussian distribution with a covariance matrix unique to every sample. Through simulated examples in which the study population is a mix of two different populations with different mean expression and patterns of coexpression, we show that BONOBO outperforms other methods in estimating sample-specific coexpression even when the underlying assumption of multivariate normality is violated, thus making the method adaptable to a wide range of applications.

In developing BONOBO, we also assumed that the covariance matrix for a particular individual follows an inverse-Wishart distribution, which is the multivariate analog of inverse-gamma, the most widely used prior distribution for the univariate variance parameter. The prior mean of the individual's covariance is assumed to be equal to the population covariance matrix estimated using all other samples in the data, which is an unbiased estimate of the population covariance. In BONOBO, the prior degrees of freedom, which characterize how much the individual covariance might vary from the population covariance, is estimated from the data empirically to capture how different the individual of interest is from the rest of the population; if that individual is an outlier, its covariance is allowed to deviate further from the population covariance. If the individual is more similar to the rest of the population, its covariance is assumed to deviate less from the population covariance. Consequently, the assumption of an inverse-Wishart prior distribution on the individual-specific covariance matrices is not too stringent for practical purposes.

It is important to note that benchmarking single-sample networks is particularly challenging (De Marzio et al. 2023). Because our goal is to estimate sample-specific coexpression, we simulated multiple gene expression samples for every individual from a multivariate normal distribution and used these samples to calculate individual-specific coexpression matrices. We showed that, for these data, the MSE of the sample-specific coexpression networks estimated by BONOBO was much lower than that of the other methods. Further, as the dimensionality of the estimation problem increased, BONOBO provided increasingly better performance than its closest competitor, SWEET. One potential reason could be that BONOBO assumes that individual-specific coexpression matrices follow an inverse-Wishart distribution with a prior mean equal to the coexpression computed from all other samples in the data set, implying that individual-specific coexpression is a priori assumed to be centered around the population's aggregate coexpression pattern. Intuitively, this is a reasonable assumption as a sample-specific coexpression network should only deviate slightly from an aggregate coexpression network.

However, assuming that the sample-specific correlation is centered around the aggregate correlation may also impact our results by reducing the overall variability of the estimated networks. When we applied BONOBO to the yeast data both for the cell cycle and the TF KO perturbation experiments, we found high values of correlation (with pairwise correlation > 0.99 in most cases) across all samples. This could present problems when using BONOBO to detect outlying samples or when using it in highly heterogeneous populations. In these situations, other methods, such as LIONESS::Pearson, might be more appropriate for identifying samples that are distant from the aggregate estimate.

Methodologically, this situation can be mitigated by imposing a noninformative prior on the individual-specific coexpression matrix, such as the Lewandowski–Kurowicka–Joe distribution with parameter 1 (*LKJ*(1)) (Lewandowski et al. 2009), which puts uniform probability over the space of all correlation matrices. However, a completely noninformative prior would not allow BONOBO to borrow information from other samples. A possible trade-off would be a mixture prior distribution ω*LKJ*(1) + (1 − ω)*InvWishart*(*V*_*i*_, ν_*i*_), where *InvWishart*((ν − *g* − 1)*S*_*i*_, ν_*i*_) is the prior used by BONOBO, and 0 < ω < 1 is a mixture weight that captures how far the outlier is from the center of the population. This prior specification is not conjugate to multivariate normal covariance, and therefore, the posterior distribution would not have a closed-form expression, increasing the computational complexity of the estimation algorithm.

We also note that BONOBO has some intrinsic limitations. Although we applied BONOBO exclusively to transcriptomic data, the model could be adapted to other omic data types (such as epigenomics or proteomics) with minor modifications (provided the data can be suitably transformed to resemble a unimodal distribution). However, it is important to note that BONOBO is not applicable for omic modalities characterized by binary or categorical data, such as mutation profiles. A potential area for future research could involve extending our model to incorporate interactions across a broader range of omic data types through hierarchical latent variable models or association measures other than the Pearson correlation coefficient. Extending BONOBO to other correlation measures, such as the Spearman's rank correlation coefficient, would also allow us to overcome the limitations intrinsic to the Pearson correlation coefficient, such as the fact that it is known to be heavily influenced by outliers. Extending BONOBO to other measures, such as Chatteerjee's correlation coefficient (Chatterjee 2021), could allow BONOBO to estimate networks based on nonlinear associations between the expression of genes.

Finally, we recognize that in its current implementation, BONOBO does not control for false-discovery rate (FDR) when computing the *P*-values for specific edges in individual coexpression networks. Theoretically, by including an edge with *P*-value bounded above by α, BONOBO includes the expected α*g*(*g* − 1)/2 false-positive edges in the network, where *g* denotes the number of genes. We believe the inclusion of false-positive edges might be problematic, although to what extent BONOBO might be prone to false discovery is hard to verify owing to the absence of ground truth for real data applications. In the next release of BONOBO, we plan to implement a Benjamini–Hochberg procedure for FDR correction and give users the option to choose between raw *P*-values and adjusted *P*-values.

### Software availability

BONOBO is available through the Network Zoo package (netZooPy v0.10.0; https://netzoo.github.io); specific code can be found inside the https://github.com/netZoo/netZooPy/tree/master/netZooPy/bonobo folder. Data and code to reproduce the simulation experiments and real data analysis are available at the Harvard Dataverse (https://dataverse.harvard.edu/dataset.xhtml?persistentId=doi:10.7910/DVN/CAVAAB). We have also included a copy of the code in the Supplemental Material of this paper (Supplemental Code). A tutorial on generating and analyzing BONOBO networks on yeast is available on Netbooks (version 2.3.4) (Ben Guebila et al. 2022b; https://netbooks.networkmedicine.org/) under the title Estimating Single-Sample Coexpression Networks for Yeast Genetic Screens Using BONOBO. Finally, BONOBO networks for the breast cancer miRNA application and single-sample PANDAs for thyroid samples are accessible on GRAND (version 1.6) (Ben Guebila et al. 2021a; https://grand.networkmedicine.org/).

## Supplementary Material

Supplement 1

Supplement 2
