# Supplementary material for: Bayesian inference of sample-specific coexpression networks
Source: Genome Res. 2024 Sep;34(9):1397–410. doi: 10.1101/gr.279117.124 (PMC11529861; doi:10.1101/gr.279117.124)
Supplement: Supplement 1 [file Supplemental_material.pdf]

Supplementary Materials for: Bayesian inference of sample-specific co-expression networks

Enakshi Saha[1,*], Viola Fanfani[1,*], Panagiotis Mandros[1], Marouen Ben-Guebila[1], Jonas Fischer[1], Katherine Hoff-Shutta[1,2], Dawn L. DeMeo[2,3], Camila Lopes-Ramos[1,2,3], and John Quackenbush[1,2,4,**]

[1]Department of Biostatistics, Harvard T.H. Chan School of Public Health, Boston, MA
[2]Channing Division of Network Medicine, Brigham and Women's Hospital, Boston, MA, USA
[3]Department of Medicine, Harvard Medical School, Boston, MA, USA
[4]Department of Data Science, Dana-Farber Cancer Institute, Boston, MA, USA
[*]These authors contributed equally.
[**]Corresponding author: johnq@hsph.harvard.edu

July 26, 2024

Contents

S1 Supplementary Materials and Methods **2**
S1.1 Proofs of Theoretical results . . . 2
S1.1.1 Proof of Theorem 1 . . . 2
S1.2 Simulation Experiments . . . 2
S1.2.1 Simulation Example 1: varying sample size . . . 2
S1.2.2 Simulated Example 2: mixture population . . . 3
S1.2.3 Simulated Example 3: varying number of genes for homogeneous population . . . 3
S1.2.4 Simulated Example 4: varying the number of genes for mixture population . . . 3
S1.2.5 Simulation Example 5: gene deletion . . . 4
S1.2.6 Simulation Example 6: varying degrees of freedom . . . 4
S1.3 Computational Resources . . . 5
S1.4 Yeast Cell Cycle Data . . . 6
S1.5 Yeast Perturbation Dataset . . . 6
S1.5.1 Comparison with other methods . . . 7
S1.6 miRNA-mRNA co-expression in breast cancer subtypes . . . 8
S1.6.1 Data preprocessing . . . 8
S1.6.2 Differential Coexpression Analysis . . . 8
S1.7 Sex-differences in thyroid gene expression . . . 9
S1.7.1 Preparing Gene Expression Data for Analysis . . . 9
S1.7.2 Designing a Sex-specific Transcription Factor-Gene Motif Prior . . . 9
S1.7.3 Designing the Protein-protein Interaction Prior . . . 10
S1.7.4 Differential Targeting Analysis using Sample-specific Regulatory Networks . . . 10
S1.7.5 Pathway Analysis of differentially targeted genes in thyroid samples . . . 10
S1.8 Normality of gene expression . . . 10

S2 Supplementary Tables **30**

S1 Supplementary Materials and Methods

S1.1 Proofs of Theoretical results

S1.1.1 Proof of Theorem 1

Under assumption (1), the conditional likelihood of the centered expression of the i-th sample can be written as

$$\mathbb{P}(x_i - \bar{x}|V_i, \{e_j\}_{j\neq i}) \propto |V_i|^{-1/2} \exp\left(\frac{1}{2}(x_i - \bar{x})^T V_i^{-1}(x_i - \bar{x})\right) \tag{1}$$

Under assumption (2), given all other samples in the data, the prior probability density of the covariance matrix of the i-th sample can be written as

$$\mathbb{P}(V_i|\{x_j\}_{j\neq i}) \propto |V_i|^{-(\nu_i+g+1)/2} \exp\left(\frac{1}{2} trace\left((\nu_i - g - 1)S_i V_i^{-1}\right)\right) \tag{2}$$

Combining 1 and 2, the posterior distribution of V_i can be written as

$$\begin{aligned}\mathbb{P}(V_i|\{x_i\} \cup \{x_j\}_{j\neq i}) &\propto \mathbb{P}(x_i - \bar{x}|V_i, \{x_j\}_{j\neq i})\mathbb{P}(V_i|\{x_j\}_{j\neq i}) \\ &\propto |V_i|^{-(\nu_i+g+1+1)/2} \exp\left(\frac{1}{2}\left[(x_i - \bar{x})^T V_i^{-1}(x_i - \bar{x}) + trace\,(\nu_i - g - 1)\, S_i V_i^{-1}\right]\right) \\ &\propto |V_i|^{-(\nu_i+g+1+1)/2} \exp\left(\frac{1}{2} trace\left(\left[(x_i - \bar{x})^{-1}(x_i - \bar{x})^T + (\nu_i - g - 1)S_i\right] V_i^{-1}\right)\right)\end{aligned}$$

From the above expression we see that $V_i \sim InvWishart\left((\nu_i - g)\Sigma_i, \nu_i + 1\right)$, proving Theorem 1.

S1.2 Simulation Experiments

We compared BONOBO with LIONESS, SPCC, and SWEET through five simulation experiments. For each of the methods, the estimated sample-specific co-expression networks were compared with the true co-expression. We computed the mean squared error (MSE) across all gene pairs. We repeat each simulation experiment for 100 iterations (100 different simulated datasets). Because LIONESS and SPCC do not bound the network edge weights to values between -1 and 1, the resulting MSE values are much higher than in BONOBO. Consequently, for LIONESS and SPCC, we scaled the off-diagonal elements of each estimated co-expression matrix by the maximum of the absolute values of all the off-diagonal elements, and set the diagonal entries to one to resemble the Pearson correlation matrices produced by BONOBO, thus providing a more “fair” comparison.

S1.2.1 Simulation Example 1: varying sample size

We selected the top 50 most variable and top 50 least variable genes from the GTEx thyroid samples. For this set of 100 genes, we computed the mean gene expression (denoted by μ_{100}) and the covariance matrix of gene expression (denoted by Σ_{100}). We then simulated the gene expression distribution of these 100 genes for N (ranging from 10 to 1000) individuals (samples) as follows: for every individual (sample), we simulated 100 independent observations from a 100-dimensional multivariate normal distribution with mean μ_{100} and covariance matrix Σ_{100}. The correlation matrix computed from these 100 observations was treated as ground truth for the gene co-expression corresponding to this individual. The mean of these 100 observations was then treated as a representative sample for the gene expression of that individual to be used as input to BONOBO and other methods. We implemented BONOBO, LIONESS::Pearson, SPCC, and SWEET on the resulting N gene expression samples to estimate sample-specific gene co-expression networks.

We found (Figure 2A in the main manuscript) that as sample size increased from $N = 10$ to $N = 1000$, the average MSE computed over 100 iterations of the simulation experiment decreased rapidly for both BONOBO and SWEET and finally stabilized for sample size greater than 50. For LIONESS::Pearson and SPCC, the average MSE did not vary much over different sample sizes. Moreover, irrespective of the sample size chosen, the mean MSE for BONOBO was smaller than the other methods, with BONOBO providing up to 40% decrease in MSE over its closest competitor SWEET (Figure S1 top left).

S1.2.2 Simulated Example 2: mixture population

In this example, we constructed a sample of 100 individuals, $p\%$ (p ranging from 0 to 50) of which come from one population, and the remaining $(100 - p)\%$ come from a different population constructed as follows. We considered the same 100 genes as in Simulation Example 1 (section S1.2.1) from the GTEx thyroid samples. Then the GTEx thyroid samples were randomly partitioned into two groups so that one group contained $p\%$ of the samples and the other group contained the remaining $(100-p)\%$. We computed the mean gene expression of the 100 genes for each of the two partitions, denoted by μ_1 and μ_2. We generated 100 random partitions and chose the partition with the highest Euclidean distance between the two means $(\mu_1 - \mu_2)^2$. The mean gene expressions μ_1 and μ_2 and the covariances of gene expression, denoted by Σ_1 and Σ_2 for these two partitions, were then used as the parameters for the two subpopulations in the simulation.

For each of the $p\%$ of samples, we simulated 100 observations from multivariate normal with mean μ_1 and covariance matrix Σ_1. For each of the remaining $(100 - p)\%$ of samples, we simulated 100 observations from multivariate normal with mean μ_2 and covariance matrix Σ_2. Finally, for every individual, the correlation matrix computed from the simulated 100 observations was treated as the ground truth for the gene co-expression corresponding to that individual. The mean of these 100 observations was then treated as a representative sample for the gene expression of that individual to be used as input to BONOBO, LIONESS::Pearson, SPCC, and SWEET.

From Figure 2B in the main manuscript, we see that as we increased p from 0 (all samples generated from the same population) to 50 (equal number of samples generated from the two populations), irrespective of the value of p, the mean MSE for BONOBO was smaller than LIONESS::Pearson, SPCC, and SWEET (Figure S1 top right).

S1.2.3 Simulated Example 3: varying number of genes for homogeneous population

We generated $N = 100$ individual gene expression samples for varying numbers of genes (G = $100, \ldots, 1500$) as follows. We selected the top $G/2$ most variable and top $G/2$ least variable genes from the GTEx thyroid samples. For this set of G genes, we computed the mean gene expression (denoted by μ_G) and the covariance matrix of gene expression (denoted by Σ_G). Then we simulated the gene expression distribution of these G genes for 100 individuals (samples) by following the same procedure as described in Simulation Example 1 (section S1.2.1). From the average MSE (and standard deviation of MSE over 100 iterations) computed over 100 iterations (given in Table S2.1 for each value of G) for the four methods, we found that irrespective of the number of genes, average MSE for BONOBO estimates were smaller than those for LIONESS::Pearson, SPCC, and SWEET.

We also found that the mean MSE for both LIONESS::Pearson and SPCC decreased as the number of genes increased. However, upon closer inspection, this decrease in MSE was driven by the fact that as more genes were included, the new gene pairs had lower correlation values on average than the correlations in the smaller gene sets, as evidenced by the mean correlation values for number of genes $= 100, 250, 500, 750, 1000$ and 1500 recorded in the last column of Table S2.1. So, we divided the MSE by the variance of correlation for each simulation and recorded the scaled MSE in parentheses in Table S2.1. To make the results comparable across different numbers of genes, we compared the scaled MSE (MSE divided by the variance) instead of the raw MSE. We found that the scaled MSE increased for all four methods (Figure 2C) as the number of genes increased. For any fixed number of genes, however, the scaled MSE for BONOBO was much smaller than for any of the alternate methods, meaning BONOBO provided better estimates of sample-specific co-expression matrices. In particular, as we increased the number of genes from 100 to 1500, the difference between the MSE of BONOBO and the MSE of SWEET increased noticeably (Figure S1 bottom left).

S1.2.4 Simulated Example 4: varying the number of genes for mixture population

We generated $N = 100$ individual gene expression samples for $G(= 100, \ldots, 1500)$ genes. For any fixed number of genes, we constructed a sample of 100 individuals, 20% of whom come from one population with the remaining 80% coming from a different population; the sampling procedure is described in section S1.2.2 above.

Here, we also found for all methods that the scaled MSE increased with the number of genes (Figure 2D). Once again, for any fixed number of genes, BONOBO performed considerably better than the other methods. Furthermore, as the total number of genes increased from 100 to 1500, the difference between the MSE of BONOBO and the MSE of SWEET increased such that SWEET's performance declined more rapidly than that of BONOBO (Figure S1 bottom right).

Because the results from SWEET approach those of BONOBO, it is useful to compare these methods more closely. We examined the percentage decrease in MSE for these two methods as a function of the number of samples, the mixture proportion, and the number of genes (Figure S1). We found that the MSE for BONOBO had a greater and more consistent decline than SWEET across sample sizes, mixture proportions of subpopulations, and number of genes.

Overall, these simulation studies demonstrate that BONOBO outperforms both LIONESS::Pearson and SPCC by a wide margin across all parameters. While BONOBO and SWEET provide comparably accurate estimates of sample-specific co-expression for small numbers of genes (≈ 100), BONOBO performs increasingly better than SWEET as the number of genes increases. This suggests that BONOBO has a significant advantage in application to genome-scale networks.

S1.2.5 Simulation Example 5: gene deletion

We considered the same 100 genes as in Simulation Example 1 (section S1.2.1) from the GTEx thyroid samples and generated the gene expression distribution for 100 individuals as described in Simulation Example 1. Then we randomly selected $p\%$ of individuals and 1% of genes and set the gene expression value of the selected genes to 0 for these $p\%$ individuals while keeping the other $(100-p)\%$ individuals unaltered. For these $p\%$ of individuals, all correlation values involving the 1% selected gene would be exactly 0. Based on the mean MSE for the correlation values corresponding to the 1% of genes for the $p\%$ individuals (Figure 2C in the main manuscript), we found that for p between 5 to 90, average MSE for BONOBO computed over 100 iterations was lower than both LIONESS::Pearson and SPCC, whereas SWEET had comparable performance to BONOBO throughout. For p greater than 90, the mean MSE for all methods was comparable. Both sparse BONOBO and sparse SWEET were able to detect the deleted genes much more accurately compared to their non-sparse counterparts, LIONESS::Pearson and SPCC.

S1.2.6 Simulation Example 6: varying degrees of freedom

We performed two simulated analyses to demonstrate that computing prior degrees of freedom μ_i, separately for every sample i, using the approach described in the section "Fixing Prior Degrees of Freedom" of the main manuscript, performs well compared to assigning arbitrary fixed values of ν_i to all samples. Since ν_i is a one-to-one function of hyperparameter δ_i, we equivalently compared the performance of BONOBO over varying values of δ, where $\delta = \delta_i \forall i$.

In the first simulation (Figure S2 (left)), we generated 100 random datasets, each containing 100 samples of gene expression values of 100 genes, as described in section S1.2.1.

In the second simulation (Figure S2 (right)), we generated 100 random datasets from a mixture distribution, where 20% of the samples come from one population, and the other 80% samples come from another population with a different mean expression. The mean of the larger population remained the same as in S1.2.1, while the mean of the smaller population was 20% higher for every gene compared to the larger population. The correlation value for each pair of genes for the smaller population was set to 20% of the corresponding correlation value of the larger population. The covariance matrix of the larger population was assumed to be the same, as in section S1.2.1.

For both examples, we computed the mean sum of squared errors (MSE) over these 100 datasets for each chosen value of $\delta_i = \delta$, ranging between zero and one. We compared these MSEs with the MSE obtained from BONOBO or sparse BONOBO, using the data-derived calibrated values of δ_i. We found that in both simulation examples, the MSE for BONOBO with calibrated values of δ_i was comparable to the minimum MSE obtained by using a fixed choice of δ_i for all samples, thus demonstrating that a sample-specific value of the hyperparameters δ_i estimated by a data-driven approach performs better than assuming $\delta_i = \delta$ to be the same for all samples.

The efficacy of the data-driven estimation of δ_i can be explained as follows. The data-driven approach of calibrating the hyperparameter δ_i assigns different values to δ_i for each sample i, thus variably

weighting individual-specific evidence when estimating the individual-specific coexpression. Notice that δ_i is inversely proportional to the ratio $\frac{\sum_{k=1}^{g}(s_i^{(kk)})^2}{\sum_{k=1}^{g}\eta^{(k)}}$, the numerator of which quantifies the total variance of all gene expression estimated excluding sample i. For an individual who is an outlier with respect to gene expression, excluding that individual would decrease the overall gene expression variance, thereby giving a smaller value of $\frac{\sum_{k=1}^{g}(s_i^{(kk)})^2}{\sum_{k=1}^{g}\eta^{(k)}}$, which is equivalent to having a larger value of δ_i. Conversely, for an individual closer to the center of the population, excluding that individual would increase the overall variability of gene expression of the data, thus making the ratio $\frac{\sum_{k=1}^{g}(s_i^{(kk)})^2}{\sum_{k=1}^{g}\eta^{(k)}}$ larger and δ_i smaller. Thus the calibrated values of δ_i depend on how much each individual deviates from the center of the population. Therefore, for outlying samples, δ_i is estimated to be larger, thus reducing estimation bias. In contrast, for individuals closer to the center of the population, δ_i is estimated to be smaller, thus putting greater weight on the population-specific evidence and hence deriving more robust estimates of individual-specific coexpression.

S1.3 Computational Resources

We compared the time and memory usage of BONOBO to that of the other methods. For this purpose, we have isolated the core functions that generate all networks such as the ones described below:

Algorithm 1 Compute Performance

```
Input: Data data ∈ R^{G,S} where G = # genes, S = # samples

x ← preprocess(data)
for each i in S do
    net ← get_sample(x, i)
end for
```

In the above example each "preprocess" and "get_sample" function depends on which of the four methods (BONOBO, LIONESS, SWEET, SPCC) we used. As expected, by looking at the formulation of each method, BONOBO is more time-consuming, but more memory-efficient than LIONESS::Pearson, SWEET, and SPCC (Figure S3). BONOBO's most time-intensive step is the matrix inversion performed in each iteration; SWEET, LIONESS, and SPCC all require a memory-intensive pre-computation of a Pearson correlation matrix from all samples. However, it is worth noting that the cost of any of these methods involves more than computation alone and includes the entirety of the process used to generate and save these networks.

Indeed, keeping all sample-specific matrices in memory is an expensive operation that quickly grows with the number of samples; for a dataset with 5000 genes and 100 samples, while each sample requires only 180MB, it is easy to see that having all samples in memory would require 18GB, and for a human dataset with 10,000 genes and 500 samples one would need almost 380GB of memory. Consequently, a viable implementation would require saving each sample after it has been computed, which is instead a more time-consuming operation than the actual computation (Figure S4). Finally, we also acknowledge that sparsifying each individual-specific correlation matrix from BONOBO increases the resources required by the process. Effectively, the computation of a p-value matrix for each sample doubles the necessary time and memory (Figure S5).

The SWEET approach to sparsification involves transforming the values to z-scores, which can be inefficient. In its publicly available code, SWEET implements a naive approach in which all saved networks are first loaded in memory to compute means and standard deviations which are then used to transform values to z-scores in a second step. As explained in the above example, this is not feasible for larger datasets. One could possibly implement an iterative strategy to compute the mean and standard deviation online, and then use those values to estimate z-scores of each pre-saved sample, which would improve performance. For the purpose our analysis, we implemented a more efficient approach to sparsify all SWEET large networks that uses a composed estimator for the standard deviation.

Overall, all methods require a trade-off between time and memory, where BONOBO is more memory

efficient than the others, and it avoids expensive MCMC thanks to the closed form solution used to compute p-values.

S1.4 Yeast Cell Cycle Data

We applied BONOBO to cell-cycle-synchronized yeast microarray data (Gene Expression Omnibus: GSE4987 [14]) that had been previously normalized and pre-processed [12]. This experiment has duplicate microarray expression profiles of yeast cells through approximately two cell cycles, each consisting of 24 time points (48 samples in total); we refer to the resulting individual sample networks as "BONOBOs." The expression data shows a periodicity of transcript levels and so we expect the BONOBO networks to reflect changes in the cell-cycle phase. Indeed, by looking at the correlation between each pair of BONOBOs, we find higher values in correspondence of the same cell cycle phase across both replicates (Figure S6). Moreover, we applied the sparsification procedure for each BONOBO, with different confidence thresholds ($pval < 0.01$,0.05,0.1,0.2); while a low threshold such as $p < 0.01$ might be too restrictive and result in a low number of edges, we can see that the sparsification is reliable for values above 0.05. It is worth noting that the number of non-zero edges for each sample captures the expression signal's periodicity (Figure S6). We compared BONOBOs in different cell-cycle phases by collapsing the networks into those representing three cell-cycle phases (G1, S, G2/M) and averaging the sparsified BONOBOs in each phase to arrive at three networks, one for each cell-cycle phase. We calculated the differences in degree between each node between the phase-average networks in two sequential transitions (G1 to S and S to G2/M) and ran a Gene Set Enrichment Analysis (GSEA) on the genes ranked by degree difference. Using the Gene Ontology database (downloaded from the Molecular Signatures Database (MSigDB) (http://www.broadinstitute.org/gsea/msigdb/collections.jsp)), we found that both transitions are enriched for DNA replication processes. We also found that the G1 to S transition is enriched for positive G1/S regulation and the S/G2-M transition is enriched for negative regulation of G1/S transition (Figure S7).

S1.5 Yeast Perturbation Dataset

We downloaded scRNA-seq data from [9] representing transcriptional responses of genetically and environmentally perturbed *Saccharomyces cerevisiae* knockout (KO) strains. The knockouts include TFs in the NCR, The Nitrogen Catabolite Repression (NCR) pathway (*GAT1, GLN3, DAL80, DAL81, DAL82, and GZF3*), from the General Amino Acid Control (GAAC) (*GCN4*), from the Ssy1-Ptr3-Ssy5-sensing (SPS) pathway (*STP1, STP2*), and finally TFs from the retrograde pathway (*RTG1, RTG3*). Each of these strains was then grown in 11 media, with different carbon and nitrogen sources: Yeast Extract, Peptone, Glucose (YPD), YPD, Harvested after Post-Diauxic Shift (YPDDiauxic), YPD + 200 ng/mL Rapamycin (YPDRapa), Yeast Extract, Peptone, Ethanol (YPEtOH), Minimal Media, Glucose (MinimalGlucose), Minimal Media, Ethanol, (MinimalEtOH), Nitrogen Limited Minimal Media with Glutamine (Glutamine), Nitrogen Limited Minimal Media with Proline (Proline), Nitrogen Limited Minimal Media with NH4 (AmmoniumSulfate), Nitrogen Limited Minimal Media with Urea (Urea), Carbon Starvation (CStarve). For all strains, we have scRNA-seq expression data from which we created pseudo-bulk expression values by averaging the counts for all cells in each genotype-growth medium combination. Each sample is named to represent the gene perturbation and media condition; for instance, sample 'gcn4_ypd' refers to the sample where gene *GCN4* is knocked down and the strain is grown in YPD.

To avoid correlation inflation, we removed genes that have no counts in more than 80% of samples. In total, we generated BONOBOs for 132 samples, and we computed co-expression networks for the 5804 genes meeting our selection criteria. However, because the process of removing genes expressed at low levels might remove important TFs or condition-specific genes, we also applied a second, less stringent filter in which we removed only genes that are always zeros (those not expressed in all samples), leaving 6520 genes from which we computed a second set of 132 correlation networks. Pathway over-representation analysis is conducted with the GSEApy package [6] using the KEGG 2018 gene set.

S1.5.1 Comparison with other methods

We also applied SPCC, LIONESS::Pearson, and SWEET to the same preprocessed yeast data. For SWEET, we were also able to retrieve the z-scores and associated p-values for each network. Since the full networks are large and include many near-zero edges, we applied two sparsification strategies; for BONOBO and SWEET, we can estimate edge p-values and threshold each network for $p < 0.01$. These are called "BONOBO_sparse" and "SWEET_sparse." Since SPCC and LIONESS::Pearson do not have a strategy for computing p-values, we selected the 500 edges with the highest and lowest average edge values. For consistency, we also applied the same strategy to the SWEET and BONOBO networks; the resulting correlation networks are referred to as "BONOBO_top1k", "SWEET_top1k", "SPCC_top1k," and "LIONESS_top1k."

For each method, beyond visual inspection of correlation, we tested whether samples in the same media, or those with the same KO target, are more similar to each other than the rest of the samples. That is, whether sample 'gcn4_ypd' is more similar to any 'x_ypd' than to the samples grown in other media. To do that, we used three different strategies: 1) correlation ranking, 2) clustering performance, and 3) distance testing. First, we compute the Pearson correlation between all pairs of networks obtaining a 132x132 correlation matrix ($C^{(m)}$) for each of the six methods ($m = \{$'BONOBO_sparse', 'SWEET_sparse', and 'BONOBO_top1k', 'SWEET_top1k', 'SPCC_top1k', 'LIONESS_top1k'$\}$). Since these networks tend to be similar to each other, these values are all positive. For each sample (each row of the C(m) matrix), we expect to find samples in the same media growth conditions to have higher correlation values than the others. Hence we compute a ROC (and relative AUC) for each sample s, between the $C^{(m)}_{s,:}$ correlation values and binary label where samples in the same media are assigned a 1 label, while the other samples are assigned a 0 label. Similarly, we repeated the procedure for samples with the same genotype (Figure S10).

While AUROC performance on correlation is a good measure of network similarity, we also wanted to add a statistical testing layer to this analysis to check whether the similarity of intra-group samples is statistically higher than those with respect to the inter-group samples. To this end, we first computed the distance (Euclidean and cosine) between all pairs of networks obtaining a 132 x 132 distance matrix ($D^{(m)}$) for each of the 6 network inference methods m. For each row $D^{(m)}_{s,:}$, we expect lower values for samples in the same growth media group than for the other samples. Consequently, for each sample s, we applied a Mann-Whitney U-test with the null hypothesis being that the distance between sample s and those in the same group is the same as sample s and those in the other groups. The alternative hypothesis is that this distance is smaller. We repeat the procedure for samples in the same genotype group. In total, for all 6 network types, we tested the 132 samples twice, for either same-media or same-genotype conditions, and applied a Bonferroni correction to the p-values (Figure S11).

Lastly, we wanted to test whether the same media samples tend to cluster together. We used three different clustering algorithms in which we can select the number of clusters (k-means, Spectral, and Agglomerative clustering) and computed different clustering metrics (adjusted rand index (ARI), adjusted mutual information (AMI), completeness, and Fowlkes-Mallows). The metrics were computed against ground truth labels that group together the samples in the same growth media or with the same KO genotype, Figure S12.

We repeated the analysis on the dataset where we removed only all-zero genes (Figures S13, S15, S14). Results were largely similar, with BONOBO performing at least as well as the other methods in detecting media perturbations, compared to the other methods.

As expected from the original paper [9], and from our clustering analysis, the effects of transcription factor knock-outs are much weaker than media effects. In this context, we report the low AUROC values as a control, indicating that the networks derived by all methods are unable to detect the effects on single TF perturbations. It is important to remember that media and genetics effects overlap so that greater the AUROC values for media, the closer to 0.5 it should be for genetic perturbations. Overall, BONOBO networks for the same growth media were consistently more similar to each other than were networks for the same transcription factor knockout and the performance was generally better than those of the other network inference methods.

It is well known that benchmarking methods on real data, where there is no ground truth to compare to, is challenging at best. In this yeast dataset, we took advantage of information about the effects of perturbations on the system to test whether BONOBO was capable of recovering the "known" observation that the predominant effect in gene expression arises in response to the growth media used than to

the effect of knock-out of a single transcription factor.
However, the effect of a TF KO should be observable since one would expect the edges between that TF and its target genes would no longer exist. For example, when TF *GCN4* is knocked out, we expect the edges that were strongly correlated with it in the wild-type would undergo greater changes than those connected to any other TF in the yeast genome. For every growth medium, we first identified the top edges within the wild-type samples that are most strongly connected to each of the 10 TFs (DAL80, DAL81, DAL82, GCN4, ... (top 5% edges on the left panel of Figure S16, top 10% edges on the middle panel, and top 20% edges on the right panel). Then, for each of these edges, we used the Kolmogorov-Smirnov test to quantify whether the edge weights were significantly different between the wild-type and the KO perturbed samples.

For every TF knock-out, we computed the "true positive" (TP) rate as the proportion of edges connected to that specific TF that were significantly different between wild-type and the KO samples. We did not simply look for missing edges since a change in regulation (for example, removing an inhibitory TF) can be reflected in an edge in the gene regulatory network. The false positive (FP) rate was computed as the proportion of edges, connected to that specific TF, that are significantly different between wild-type and the KO perturbed samples when any other TF is knocked out. The false negative rate is computed similarly. Using TP, FP and FN, we compute the F1 score (= TP/TP+0.5(FP+FN)) for each method for every genotype.

We found that for every TF-KO, BONOBO has a comparable or better F1 score relative to other methods. With 20% edges, BONOBO is significantly better (p-value of one-sided KS test for BONOBO vs SWEET, LIONESS::Pearson, and SPCC are 0.0379, 0.0379 and 0.103 respectively for the analysis on 20% edges), whereas for 10% and 5% edges, LIONESS::Pearson is significantly better (p-value of KS test = 0.0233 for 10% edges and 0.0758 for 5% edges) than BONOBO, while BONOBO, SWEET, and SPCC have performance similar to each other. In these cases, it should be noted that for LIONESS::Pearson, all comparisons between edges in wild-type and genotype KO samples were always significant, leading to both TP = 1 and FP = 1, which results in a high F1 score, due to the imbalance of the groups.

S1.6 miRNA-mRNA co-expression in breast cancer subtypes

S1.6.1 Data preprocessing

Expression profiling of 489 miRNAs along with genome-wide matched mRNA profiling from 41046 probes in 101 human primary breast tumor samples was obtained from the Gene Expression Omnibus (GEO) repository (GEO accession number GSE19783 [5, 1, 8]). Expression data, along with clinical information, were downloaded from GEO using the R package GEOquery (version 2.62.2). The samples belonged to one of five breast cancer subtypes. Three samples with unspecified subtypes, along with three samples with missing subtype information, were categorized as "not classified" for downstream analysis. Thus, for downstream analysis, we consider 6 categories of subtypes: (i) Basal-like or triple negative (n = 15); (ii) ERBB2 or Her2 positive (n=17); (iii) luminal A (n=41); (iv) luminal B (n=12); (v) Normal-like (n=10) and (vi) not classified (n=6).

From the expression data, we removed mRNA and miRNA probes with missing values for all samples and unannotated miRNAs, leaving 41002 mRNA probes and 495 miRNA probes. For genes with multiple probes, we kept only the probe with the maximum variance and discarded the rest. From the 19597 unique genes, we further removed 74 Y genes and 19 unmapped genes. We constructed sample-specific correlation networks with BONOBO using the remaining 19504 genes (including 738 X genes) and 384 miRNAs. This resulted in 101 sample-specific co-expression networks, each of which constitutes correlations between pairs of genes, pairs of miRNAs, and between each gene and each miRNA.

S1.6.2 Differential Coexpression Analysis

To identify which biological pathways are most significantly associated with miRNA expression in various breast cancer subtypes, we first computed a miRNA-specific in-degree of all genes, separately

for each sample-specific BONOBO network, by summing over all edges connecting the particular gene to the miRNAs. Then we fit linear regression on the in-degree of all genes using the breast cancer subtype-specific intercepts, using the R package “limma” (version 3.50.3) [15]. For every cancer subtype, genes were ranked by the t-statistic of the “limma” model, and a gene set enrichment analysis was performed using gene sets from the Kyoto Encyclopedia of Genes and Genomes (KEGG) pathway database [10] (“c2.cp.kegg.v2022.1.Hs.symbols.gmt”). Multiple testing corrections were performed using the Benjamini-Hochberg procedure [3].

S1.7 Sex-differences in thyroid gene expression

S1.7.1 Preparing Gene Expression Data for Analysis

RNA-Seq data of 706 healthy thyroid tissue samples from the GTEx Project were downloaded from the Recount3 database [21] using the R package “recount3” (version 1.4.0). Clinical data for GTEx samples were accessed from the dbGap website (https://dbgap.ncbi.nlm.nih.gov/) under accession number phs000424.v8.p2. We removed 53 samples because they were designated as “biological outliers” in the GTEx portal for various reasons (as described in https://gtexportal.org/home/faq). The remaining 653 samples (434 males and 219 females) were used in the final analysis.

Gene expression data were normalized by Transcripts per Million (TPM), using the “getTPM” function in the Bioconductor package “recount” (version 1.20.0) [4] in the R (version 4.1.2). Lowly expressed genes (with counts ≤ 1 TPM in at least 10% of the samples) were filtered out, thus leaving 26792 (including 64 Y genes) genes for analysis. To build gene regulatory networks, we kept only those genes (26507 genes) that were present both in the filtered gene set, as well as in the TF/target motif priors (see sections S1.7.2 and S1.7.3).

We verified that the self-reported gender for the GTEx samples corresponded to the biological sex by performing a principal component analysis of gene expression values of 64 genes on the Y chromosome [Figure S22]. Since some genes on the Y chromosome were assigned expression values in females due to mismapping of some transcripts, we manually set Y chromosome gene expression values to zero for biological females.

Finally, log2-transformed TPM normalized gene expression data were used as input to BONOBO to derive sample-specific co-expression networks. These co-expression networks along with sex-specific TF/target gene regulatory prior (obtained by mapping TF motifs from the Catalog of Inferred Sequence Binding Preferences (CIS-BP) [20] to the promoter of their putative target genes) and protein-protein interaction prior (using the interaction scores from StringDb v11.5 [18] between all TFs in the regulatory prior) were used as input to PANDA to derive sample-specific gene regulatory networks, using python package netZooPy (version 0.9.10) [2].

S1.7.2 Designing a Sex-specific Transcription Factor-Gene Motif Prior

The TF-gene motif prior network produced by PANDA is a bipartite network in which edges connect transcription factors (TFs) to their target genes, with the edge values (0 or 1) indicating the presence or absence of a transcription factor motif within the promoter region of a specific target gene. To create this regulatory motif network, we downloaded transcription factor motifs for *Homo sapiens* with direct or inferred evidence from the Catalog of Inferred Sequence Binding Preferences (CIS-BP) Build 2.0, accessible at http://cisbp.ccbr.utoronto.ca. These transcription factor position weight matrices (PWM) were mapped to the human genome (hg38) using FIMO [7]. We retained only highly significant matches ($p \leq 10^{-5}$) occurring within the promoter regions of Ensembl genes (specifically, GENCODE v39 annotations retrieved from http://genome.ucsc.edu/cgi-bin/hgTables). These promoter regions were defined as the interval of [-750; +250] base pairs centered around the transcription start site (TSS). This process yielded an initial set of potential regulatory interactions involving 997 transcription factors that collectively targeted 61,485 genes.

To allow statistical comparisons between networks, we needed to ensure that male and female motif networks had identical sets of edges. Consequently, we built sex-specific transcription factor regulatory priors to account for the absence of Y chromosome genes in females. Within the female-specific regulatory prior, edges originating from or connecting to Y chromosome genes were effectively assigned a weight of zero, thus resulting in a network consisting of 52,266 edges. In a previous study,

these same sex-specific TF-gene regulatory priors were also used to identify sex differences and the effect of aging on regulatory processes associated with lung adenocarcinoma [17, 16].

S1.7.3 Designing the Protein-protein Interaction Prior

We obtained PPI data from the StringDB database (version 11.5) using the STRINGdb Bioconductor package [19]. Subsequently, we filtered the PPI data to retain only interactions between transcription factors in the TF-motif network (using a score threshold index of 0). To maintain consistency in PPI scores, we normalized them by dividing each score by 1000, thereby restricting the values to a uniform range of 0 to 1 for both the PPI dataset and the TF-motif network. Additionally, we set self-interactions between transcription factors to a value of one. Since PPI networks are inherently undirected, we transformed the data into a symmetric PPI matrix.

S1.7.4 Differential Targeting Analysis using Sample-specific Regulatory Networks

For every sample-specific gene regulatory network, we calculated the targeting score for each gene, equivalent to the gene's in-degree (defined as the sum of all incoming edge weights originating from all TFs within the network). Gene targeting scores between males and females were compared using linear regression models using the R package "limma" (version 3.50.3). The linear models accounted for the effects of relevant confounders, such as sex (Male and Female), race (White, Black or African American, Others, and Unknown), age, smoking status (ever-smoker and never-smoker), ischemic time, RNA integrity number, and batch.

S1.7.5 Pathway Analysis of differentially targeted genes in thyroid samples

We performed Gene Set Enrichment Analysis (GSEA) using the R package "fgsea" (version 1.20.0) [11] and gene sets from the Gene Ontology Biological Processes (GOBP) downloaded from the Molecular Signatures Database (MSigDB) (http://www.broadinstitute.org/gsea/msigdb/collections.jsp). Only gene sets of sizes greater than 15 and less than 500 were considered after filtering out genes not present in the expression dataset. Genes were ranked by the t-statistics produced by the limma differential targeting analysis. Multiple testing corrections were performed using the Benjamini-Hochberg procedure [3].

S1.8 Normality of gene expression

BONOBO assumes that the log-transformed bulk gene expression values follow a multivariate normal distribution. To evaluate the validity of this assumption in real life, we tested all three real datasets described in the main text for normality: the pseudobulked yeast perturbation experiment [9], the breast cancer microarray dataset[5], and RNA-sequencing expression data from thyroid tissue samples from the Genotype Tissue Expression (GTEx) Project [13] (Figure S25). Using the Kolmogorov-Smirnov (KS) test, we failed to reject the null hypothesis that expression follows a Gaussian distribution for most genes in RNA-sequencing data from GTEx thyroid (88.99% genes for females, 88.16% genes for males) and for most genes and miRNAs (84.04% of genes and microRNAs combined) in the breast cancer microarray dataset. This led us to conclude that log-transformed expression data obtained by both microarray and RNA-sequencing experiments in humans can be assumed to follow Gaussian distributions without loss of generality. In contrast, for the yeast KO pseudobulked data we were unable to reject the Gaussian distribution null hypothesis for only 66.61% of genes. Nevertheless, found that BONOBO has comparable or better performance than the other methods in the yeast KO data, thus showing good performance even when the assumption of normality is violated.

Supplementary Figures

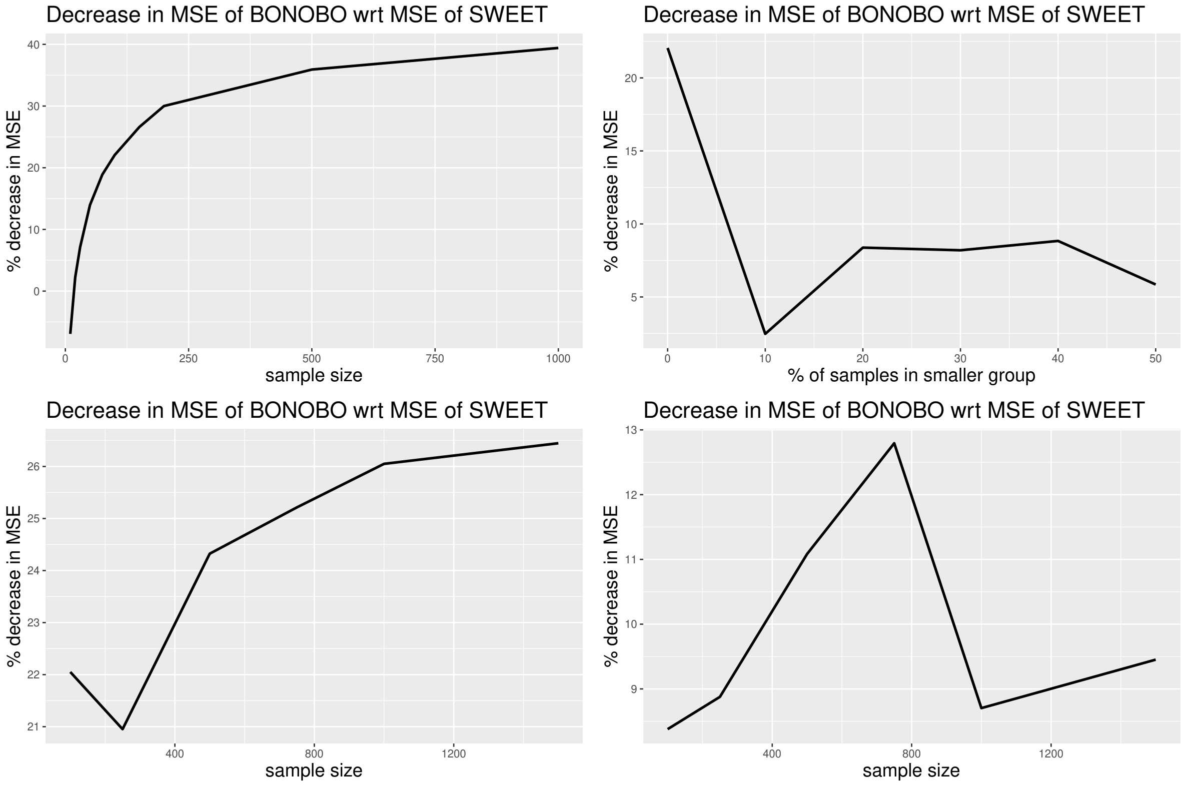

Figure S1: Decrease in mean squared error (MSE) by BONOBO, compared to MSE of SWEET for simulated data: (top left) Simulated data from homogeneous population: Change in percentage decrease in MSE with respect to sample size; (top right) simulated data from a mixture of two populations: Change in percentage decrease in MSE with respect to the percentage of samples in the smaller population; (bottom left) simulated data from homogeneous population: Change in percentage decrease in MSE with respect to the number of genes; (bottom right) simulated data from a mixture of two populations, where 20% samples come from one population and the remaining 80% samples come from another population with distinct mean and covariance of gene expression: Change in percentage decrease in MSE with respect to number of genes. BONOBO outperforms SWEET by providing lower MSE across different sample sizes, different mixture proportions of subpopulations, and different numbers of genes.

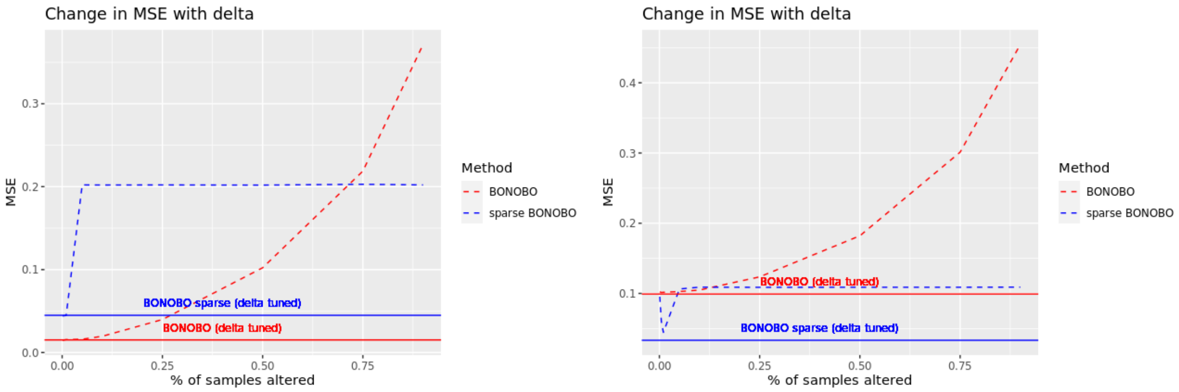

Figure S2: Comparing performance of BONOBO and sparse BONOBO fitted with fixed values of hyperparameter δ to performance with sample-specific δ values estimated by a data-driven approach: Change in MSE of BONOBO and sparse BONOBO for varying values of hyperparameter δ are shown with red and blue dashed lines, respectively. MSE for BONOBO and sparse BONOBO networks computed using δ estimated from the data are shown with red and blue solid lines respectively. (Left) Simulated data from a homogeneous population; (Right) Simulated data from a mixture of two populations. For both examples, calibrating the value of δ following the data-driven approach generally provided a smaller MSE than assigning a fixed value of δ for all samples.

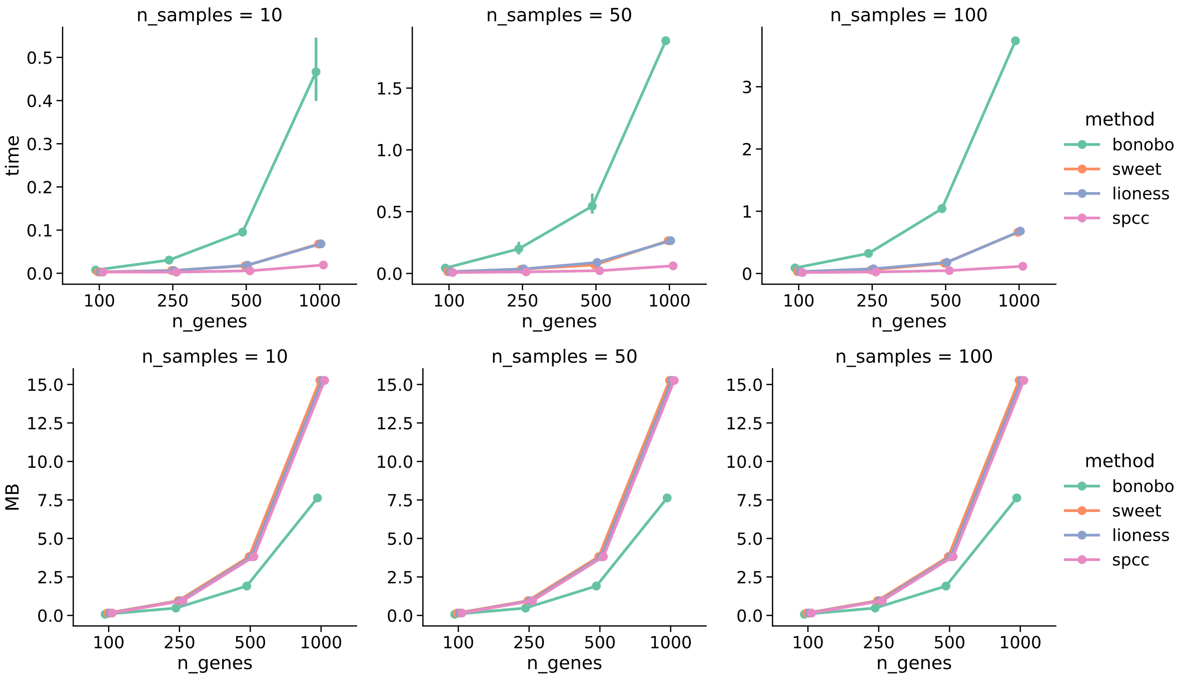

Figure S3: Computational resources required by core functions of BONOBO, SWEET, LIONESS, SPCC. Top: Time (seconds) comparison between each computation function, with respect to the number of genes and number of samples. Bottom: Memory (MB) comparison between each computation function, with respect to the number of genes and number of samples. For BONOBO, the matrix inversion step, done for each sample, is the one that requires the longest time, while all other methods are only limited by the computation of Pearson correlation matrix. Conversely, SWEET, LIONESS, and SPCC all require pre-computation and keep in memory Pearson correlation matrix from all samples, which effectively doubles the memory size required by all three methods.

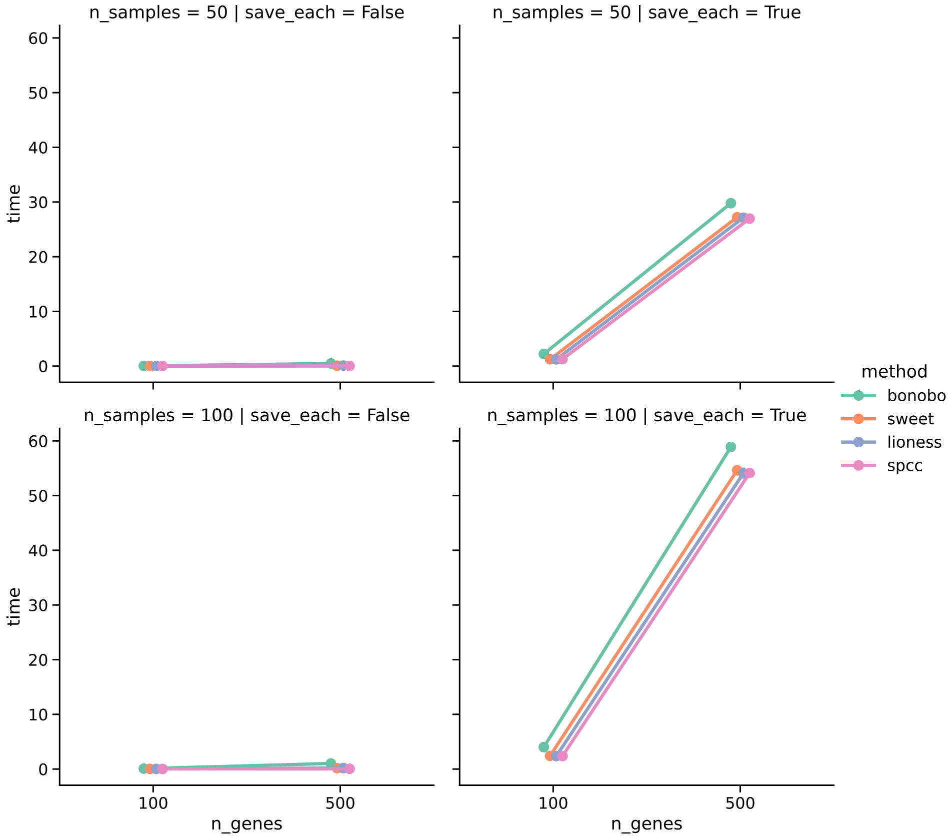

Figure S4: For each method, we compared the time (seconds) that is required to compute all samples (with different numbers of samples and genes) and the time that is required to compute and save each sample in a text format; saving to disk is much more time-consuming than the actual matrix computation. While we did not optimize this step, we wanted to highlight that the corollary steps, such as data loading and saving, are still the biggest hurdle in terms of time efficiency.

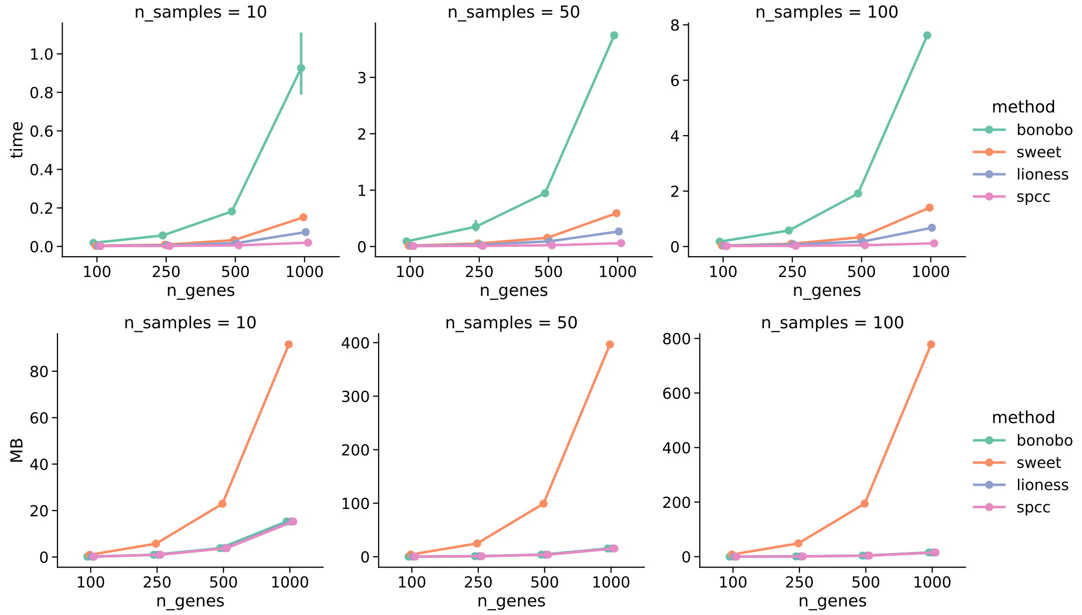

Figure S5: Computational resources required by core functions of BONOBO, SWEET with sparsification. We have kept SPCC and LIONESS:Pearsonas points of reference, although we did not apply any sparsification strategy to these methods. Top: Time (seconds) comparison between each computation function, with respect to number of genes and the number of samples. Bottom: Memory (MB) comparison between each computation function, with respect to the number of genes and number of samples. For BONOBO, the sparsification step nearly doubles both the time and memory required to carry out the entire computation. This was expected however, because for each sample, BONOBO computes a p-value matrix of the dimension of the correlation matrix. Conversely, SWEET only computes the mean and variance over all edges of all samples, which is a fast operation, but one that comes at the expense of keeping all matrices in memory. Alternately, one could implement a slower but more memory-efficient strategy to iteratively compute the mean and standard deviation at each step, which could then be used to compute z-scores of each pre-saved sample.

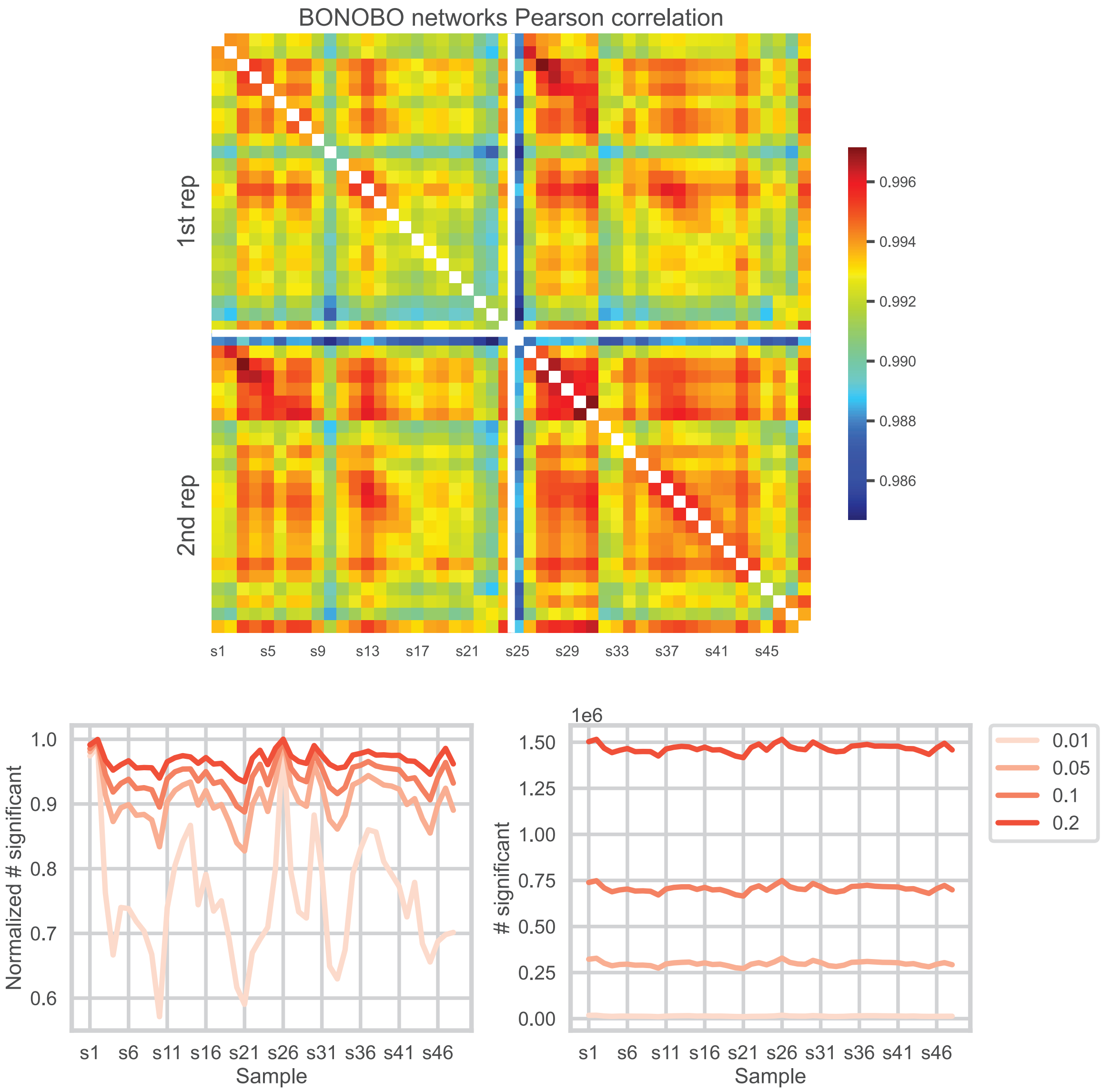

Figure S6: Top: Correlation between BONOBO network for all 48 yeast cell-cycle samples. Bottom: Proportion (left) and number (right) of non-zero edges for all sparsified BONOBO

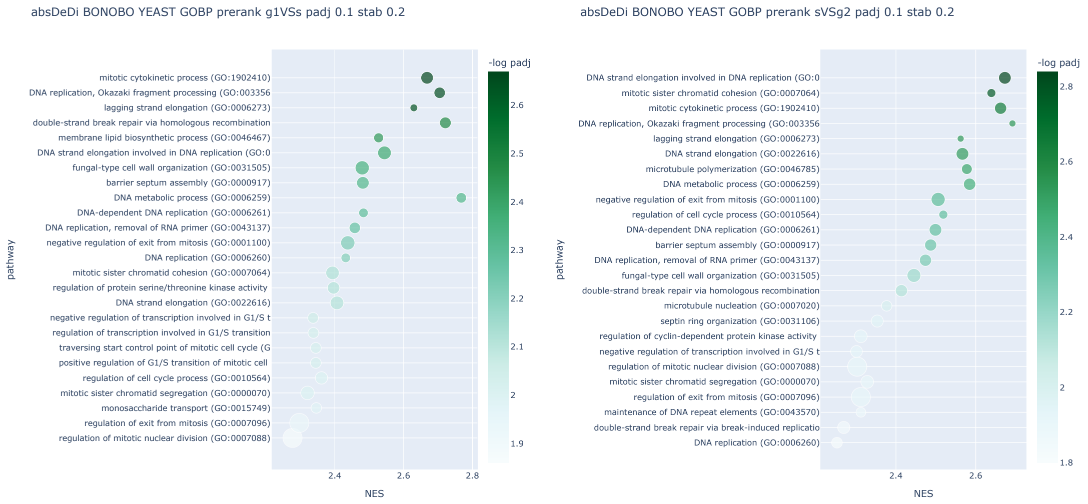

Figure S7: GSEA analysis of the G1 to S network comparison (left) and the S to G2/M networks (right). For each gene, we compute the degree, and then we compute the absolute degree difference (absDEDI) between two averaged networks. The genes ranked by absDEDI are used for the GSEA and tested for all gene ontology biological processes.

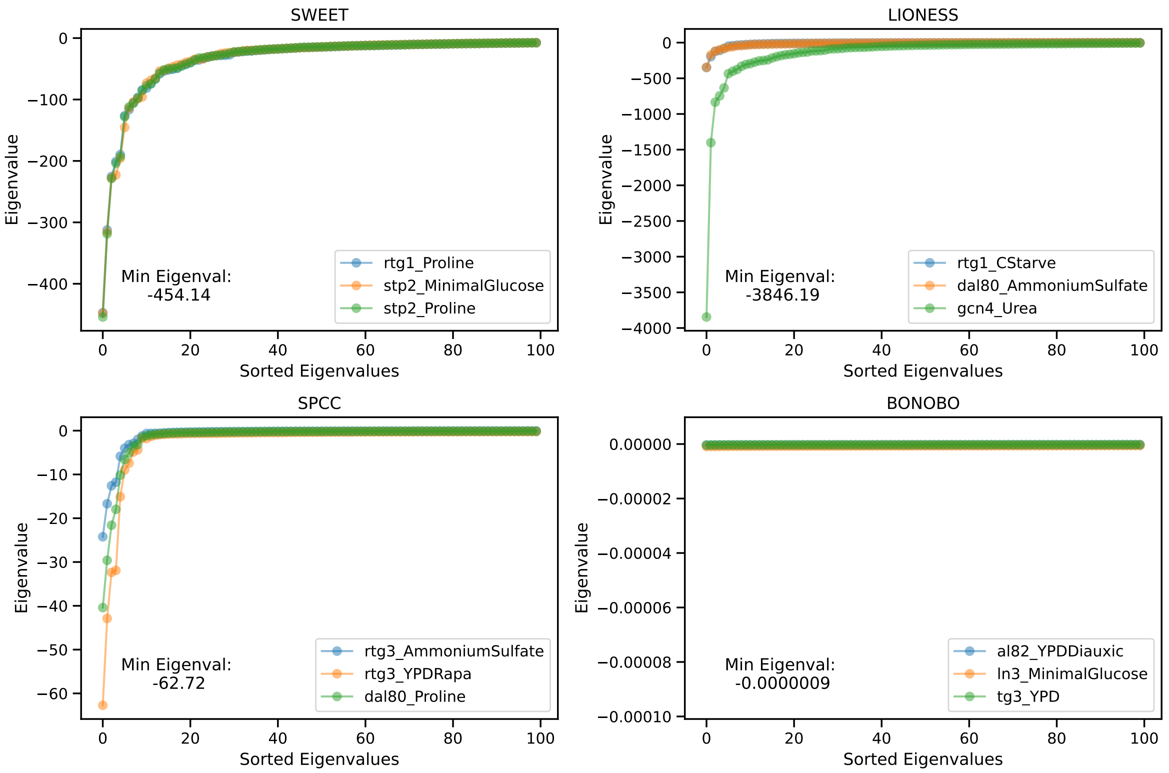

Figure S8: First 100 eigenvalues of 3 networks, chosen at random, from the yeast KO experiment computed by SWEET, LIONESS, SPCC, and BONOBO. As expected the other methods, have negative eigenvalues, while BONOBO has zero eigenvalues. In practice, for BONOBO the numerical approximation of the saved networks results in negative eigenvalues to the order of 1×10^{-7}.

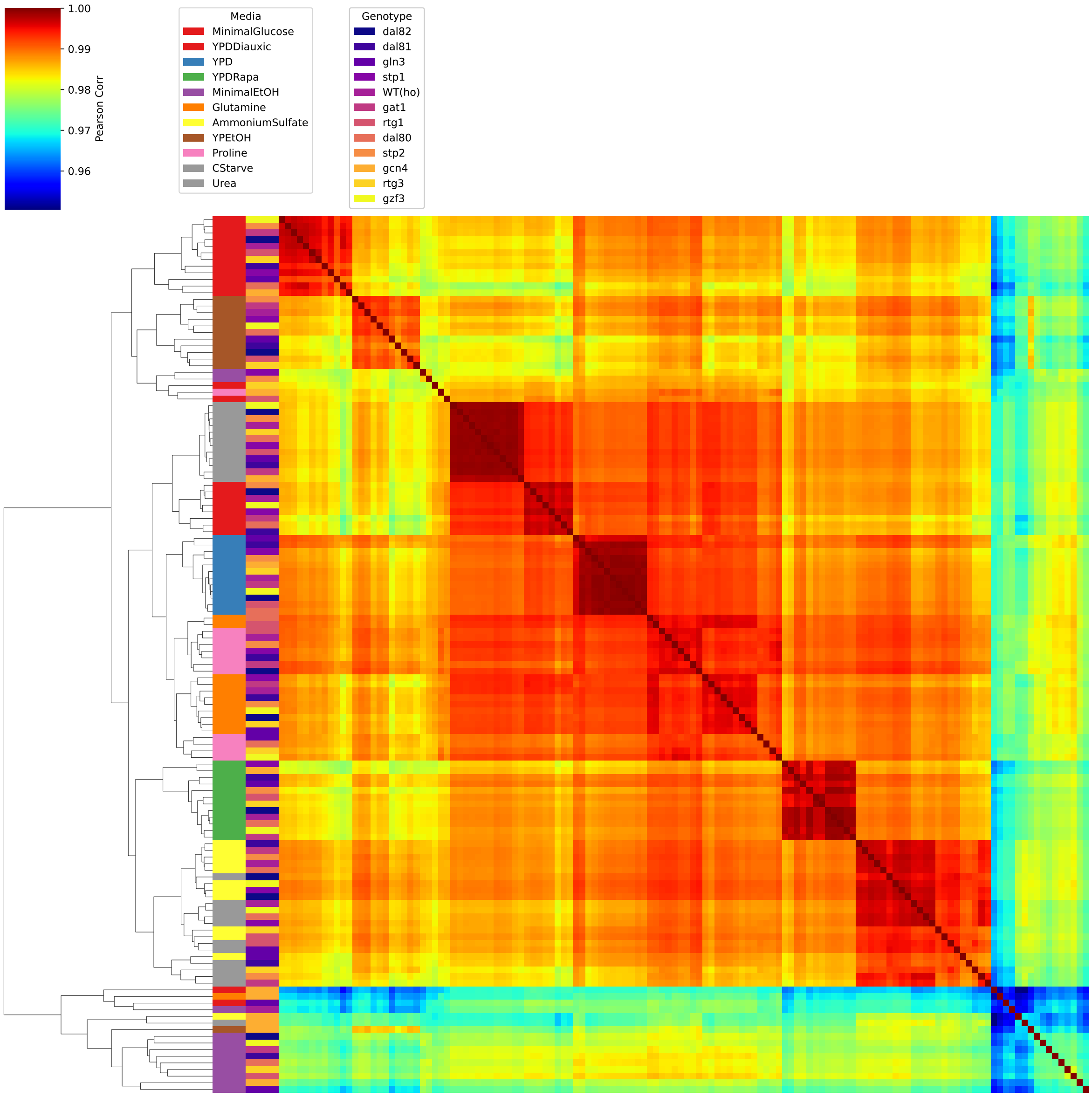

Figure S9: Pearson correlation between all sparsified BONOBOs computed on the engineered yeast data. Row labels report both the gene deletion and the growth medium for each sample. As expected, co-expression networks are more highly correlated among the strains subject to the same growth condition independent of which gene was knocked out, rather than among those with the same gene knock out, independent of growth media.

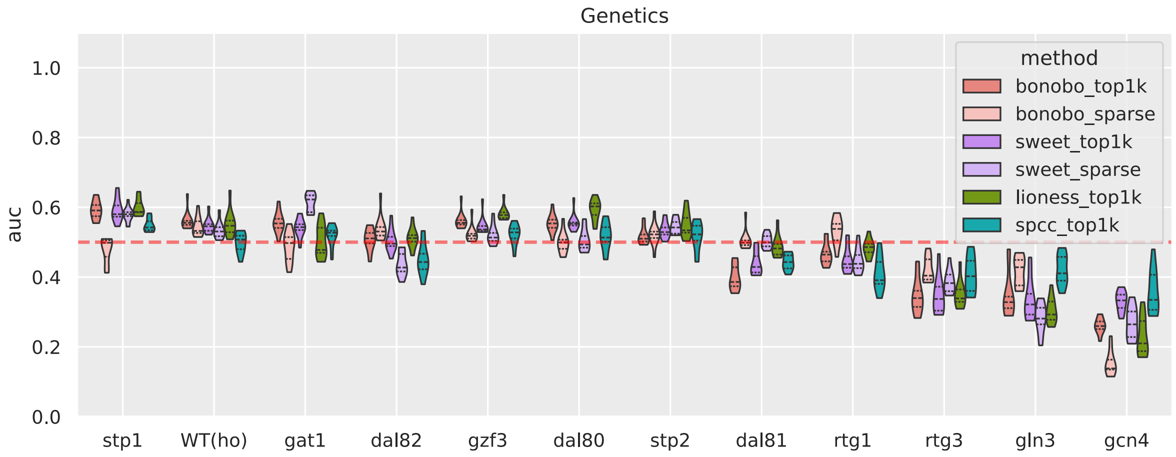

Figure S10: Network similarity between perturbed yeast samples. For each sample, we compute the AUROC values between the correlation with all the other samples and the genotype sample binary labels. $auc = 1$ means that samples in the same media have higher correlation values compared to samples in different media, $auc = 0.5$ (red dashed line) is the "random" performance, which means that samples with the same label are not more similar to each other than the rest. Networks have been inferred for the 5804 genes that have counts in at least 20% of samples.

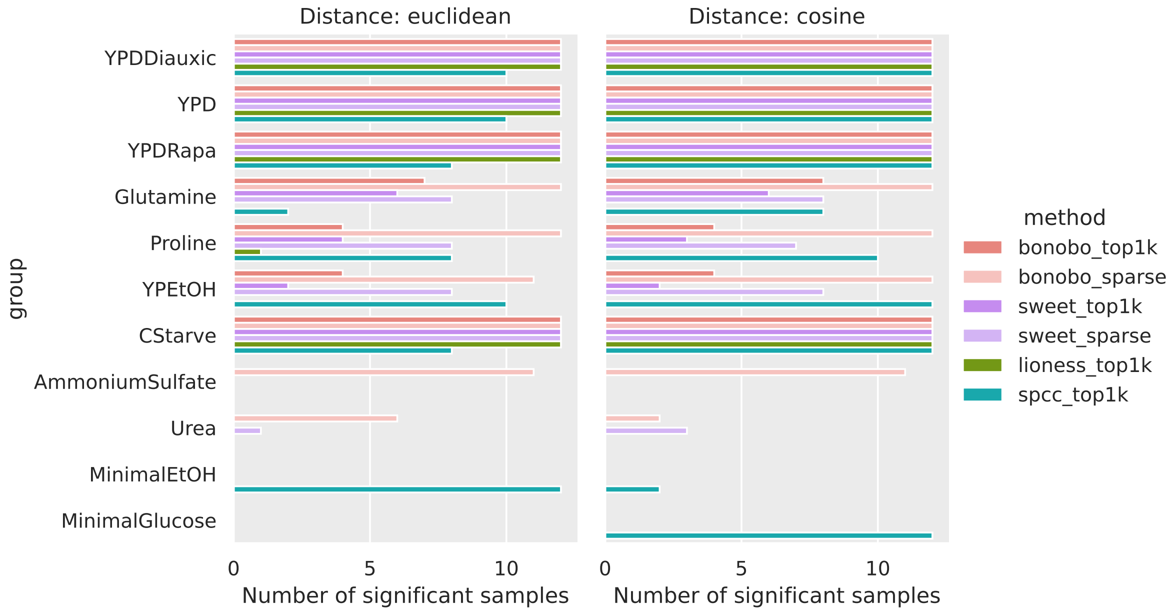

Figure S11: Number of significant (pvalue< 0.05, with Bonferroni correction) Mann-Whitney tests between the distance of samples in the same group. On the y-axis we report the groups with at least one significant test; note that no KO genotype group is significant. We denote the 6 different networks with different colors. These results are consistent with those of the correlation AUROC in Figures 3A, and we also notice that the sparsified networks seem more informative than those created by selecting the strongest edges. Note that the choice of distance metric (euclidean on the left, cosine on the right) does not substantially change the overall trend of the performance. Networks have been inferred for the 5804 genes that have RNA-seq read counts in at least 20% of samples.

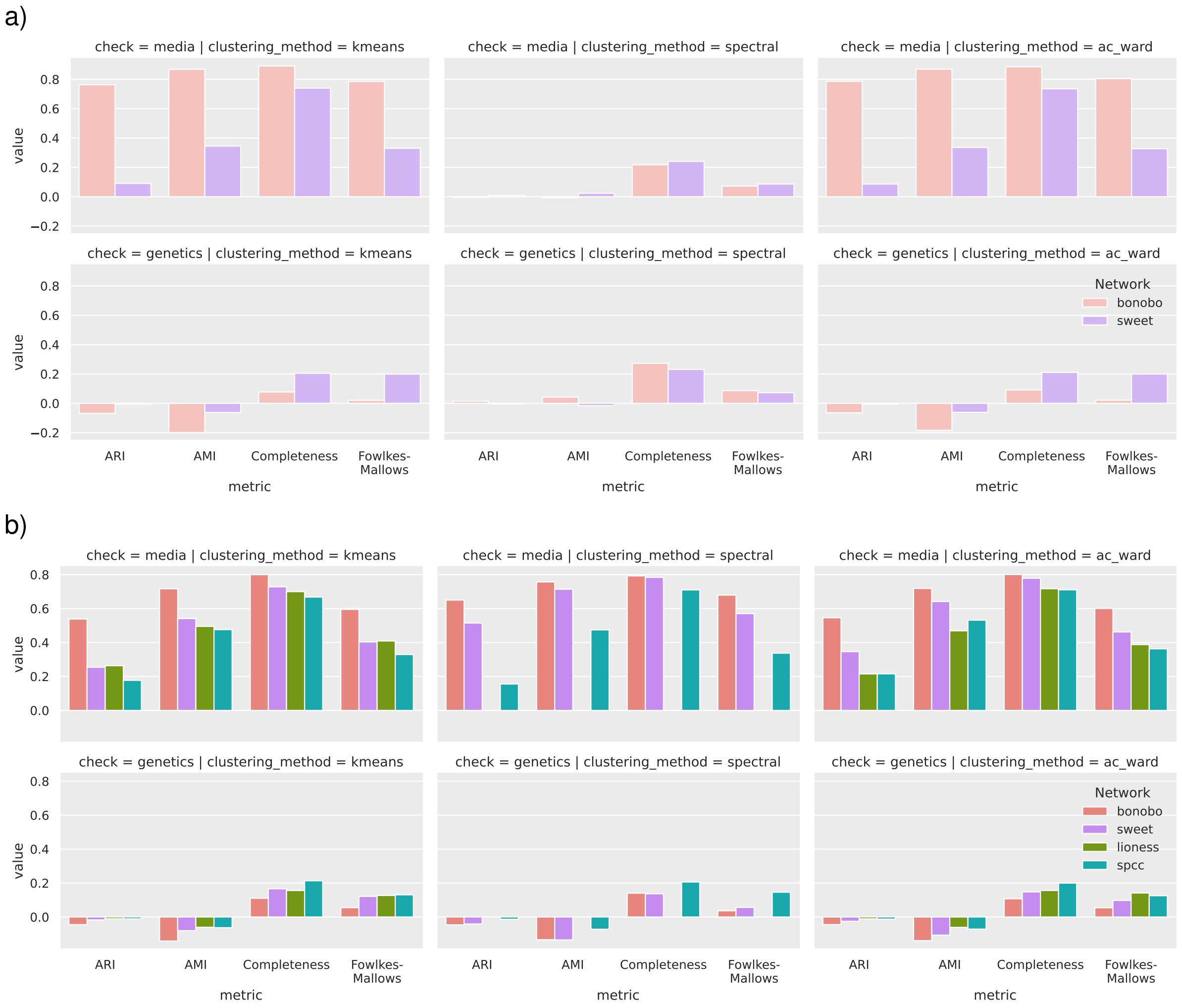

Figure S12: Clustering performance on yeast perturbation data. The top panel (a) presents the results for the sparse networks (BONOBO and SWEET), while the bottom panel (b) shows the performance of the top1k networks. For each network, we used k-means, spectral, and agglomerative clustering with Ward linkage (on columns), and we estimate the clustering performance with four different clustering scores (adjusted Rand Index (ARI), adjusted Mutual Information (AMI), Completeness, and Fowlkes-Mallows) shown are on the x-axis. The performance was evaluated against same-media labels or same-genotype (based on the KO) labels, the top and bottom row of each panel; here a higher score corresponds to clusters that better group samples from the same category. BONOBO outperforms the other methods in all conditions, showing higher scores for the same-media groups and lower values for the same-genotype groups. The top left plots (media clustering using k-means) are those shown in Figure 3B and C. Networks have been inferred for the 5804 genes that have read counts in at least 20% of samples.

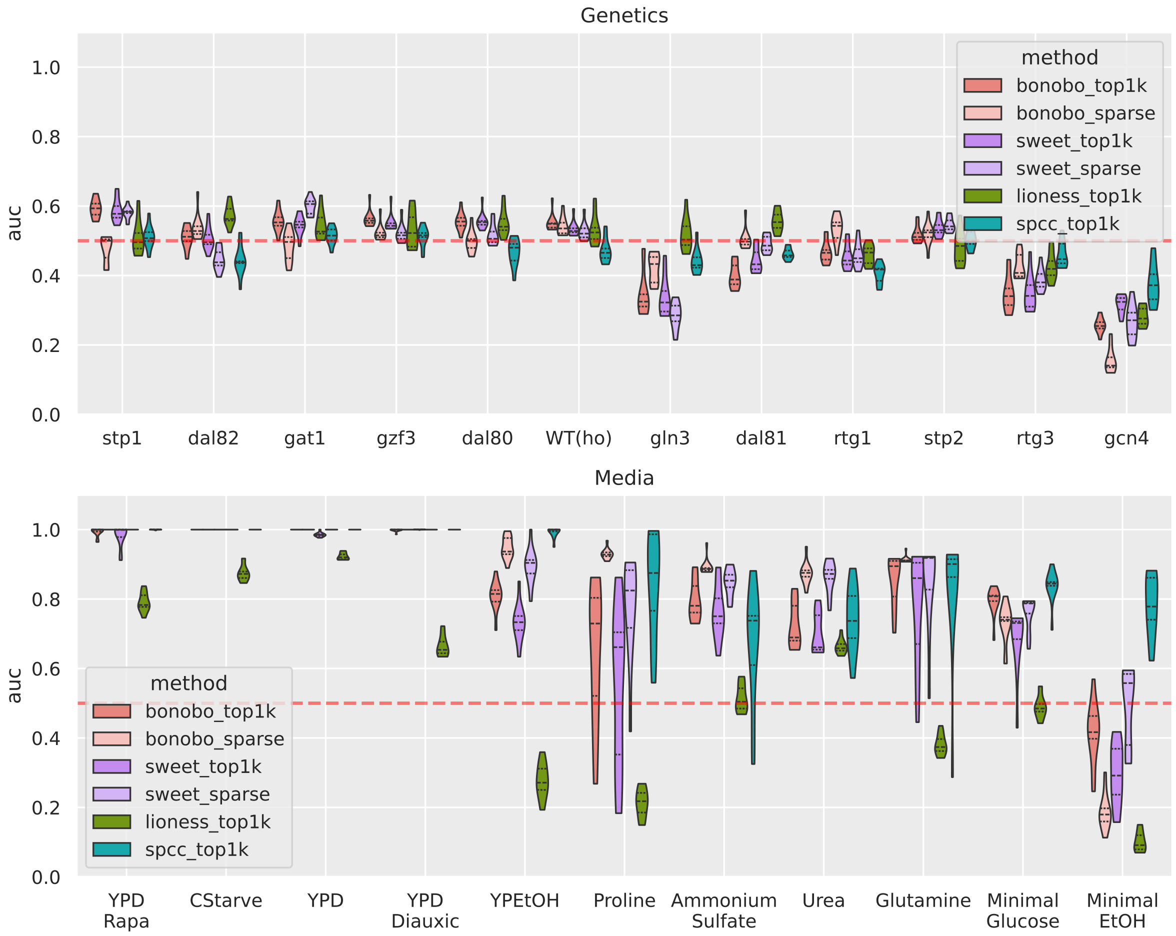

Figure S13: Network similarity between perturbed yeast samples. For each sample, we computed the AUROC values between the correlation with all the other samples and the media/genotype sample binary labels. $auc = 1$ means that samples in the same media/genotype have higher correlation values compared to samples in different media, $auc = 0.5$ (red dashed line) is the “random” performance, which means that samples with the same label are not more similar to each other than the rest. Networks have been inferred for the 6520 genes that have read counts in at least 1 sample. Results are comparable to those in Figure 3A and S10, although LIONESS:Pearson seems to suffer the presence of lowly expressed genes.

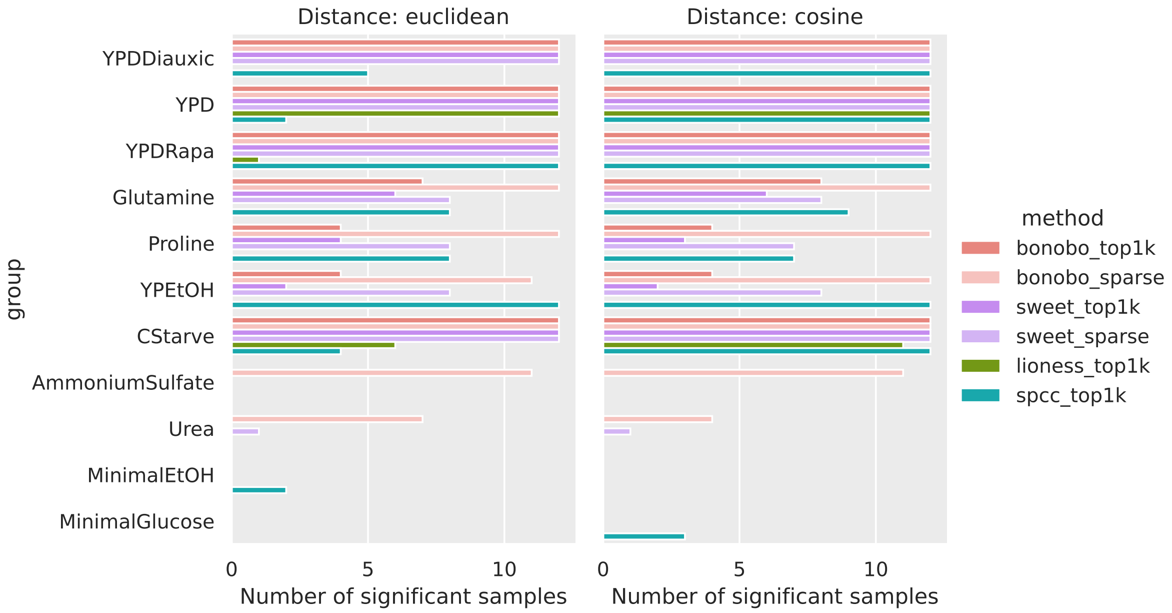

Figure S14: Number of significant (pvalue< 0.05, with Bonferroni correction) Mann-Whitney tests between the distance of samples in the same group, for the yeast perturbation dataset where we only remove all-zero genes. On the y-axis we report the groups with at least one significant test; note that no KO genotype group is significant. We denote the 6 different networks with different colors. These results are consistent with those of the correlation AUROC in Fig. S12, where BONOBO tends to outperform the other methods, and we also notice that the sparsified networks seem more informative than those created by selecting the strongest edges. Note that the choice of distance metric (euclidean on the left, cosine on the right) does not substantially change the overall trend of the performance.

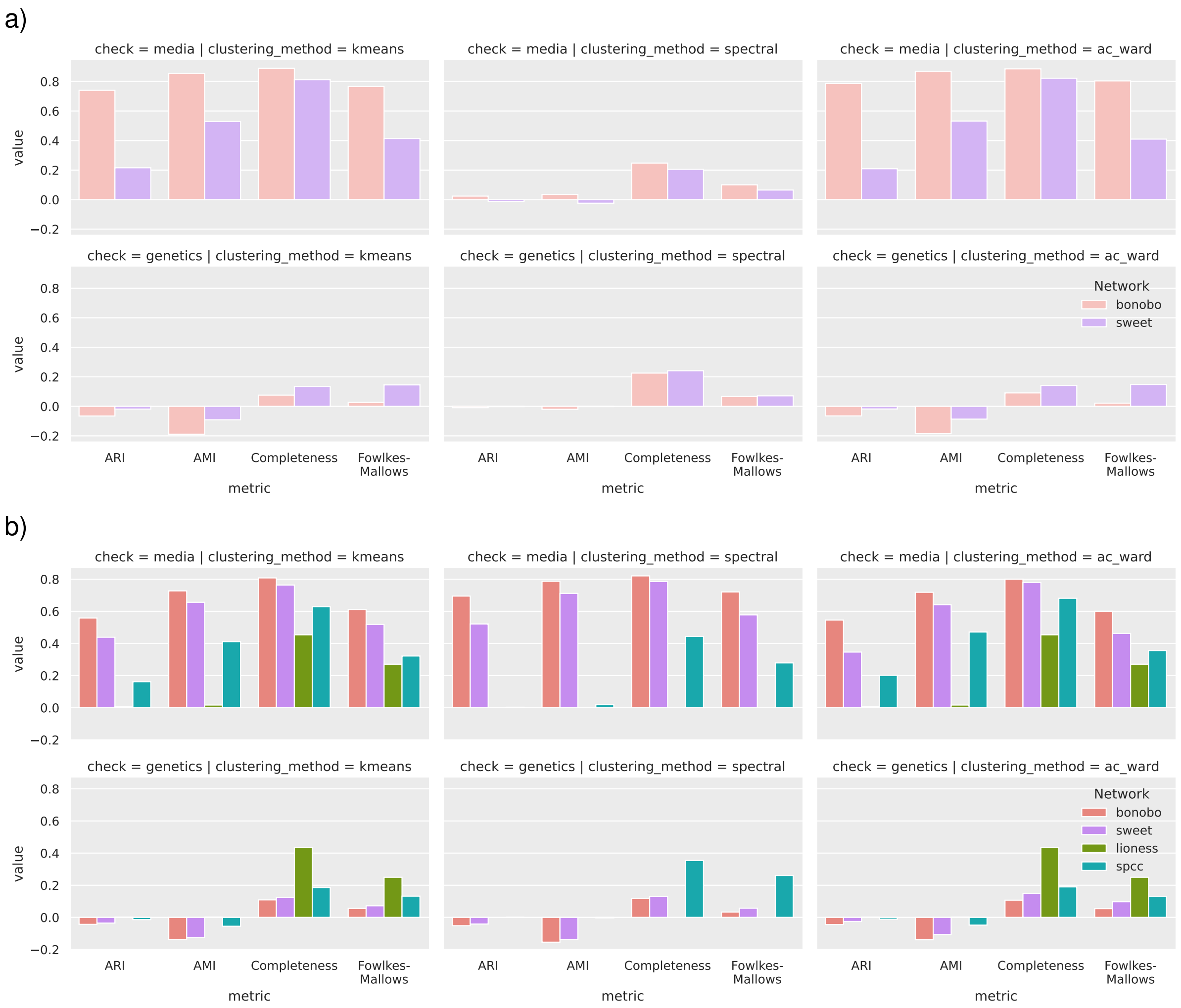

Figure S15: Clustering performance on yeast perturbation data, for the yeast perturbation dataset where we only remove all-zero genes. The top panel (a) presents the results for the sparse networks (BONOBO and SWEET), while the bottom panel (b) shows the performance of the top1k networks. For each network, we used *k*-means, spectral, and agglomerative clustering with Ward linkage (on columns) and we estimated the clustering performance with four different clustering scores (adjusted Rand Index (ARI), adjusted Mutual Information (AMI), Completeness, and Fowlkes-Mallows) shown on the x-axis. The performance was evaluated against same-media labels or same-genotype labels, top and bottom row of each panel, that is higher scores correspond to clusters that better group together samples in the same category. BONOBO outperforms the other methods in all conditions, showing higher scores for the same-media groups and lower values for the same-genotype groups.

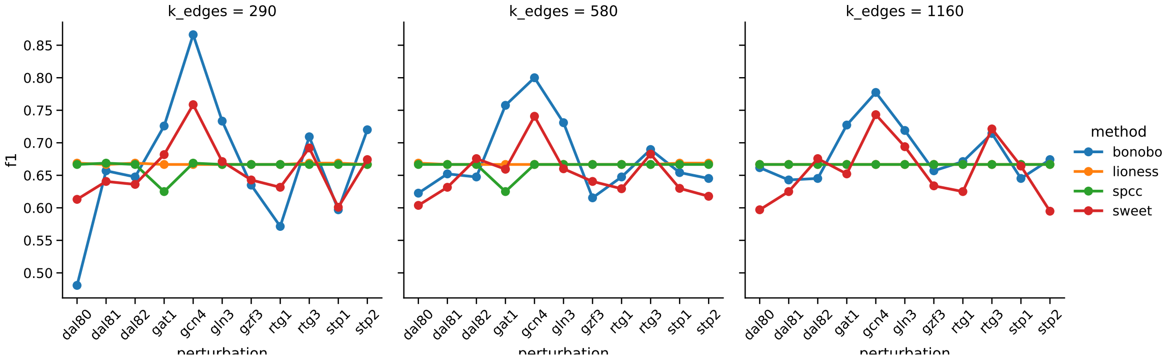

Figure S16: Comparison between BONOBO, LIONESS::Pearson, SPCC, and SWEET based on F1 score, computed based on top 5% (left), 10% (middle), and 20% strongest edges connected to the corresponding TF (denoted in the x-axis) in wild-type samples. BONOBO outperforms other methods by providing higher or comparable F1 score across all genotypes.

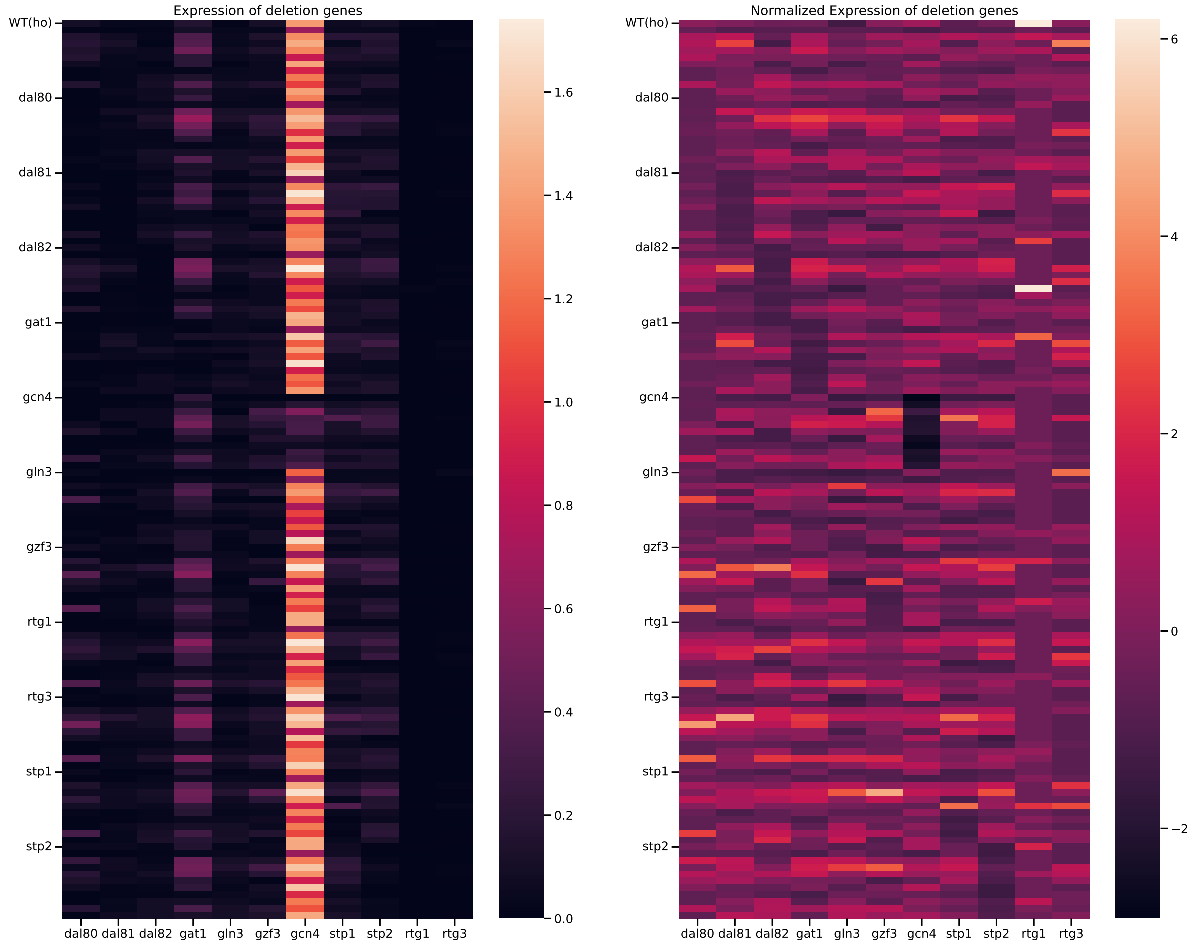

Figure S17: Expression of the perturbed TFs (columns) in each of the strains sorted by TF KO strain (rows). To avoid cluttering, instead of reporting all names on the rows, we show only the name of the genetic KO perturbation for the first sample of each group and we hide the media name. On the left, we have raw pseudobulk counts; on the right, we have normalized the values by columns; We can see that *GCN4* is on average the most expressed TF, while also being the one for which the effect of deletion is better visible.

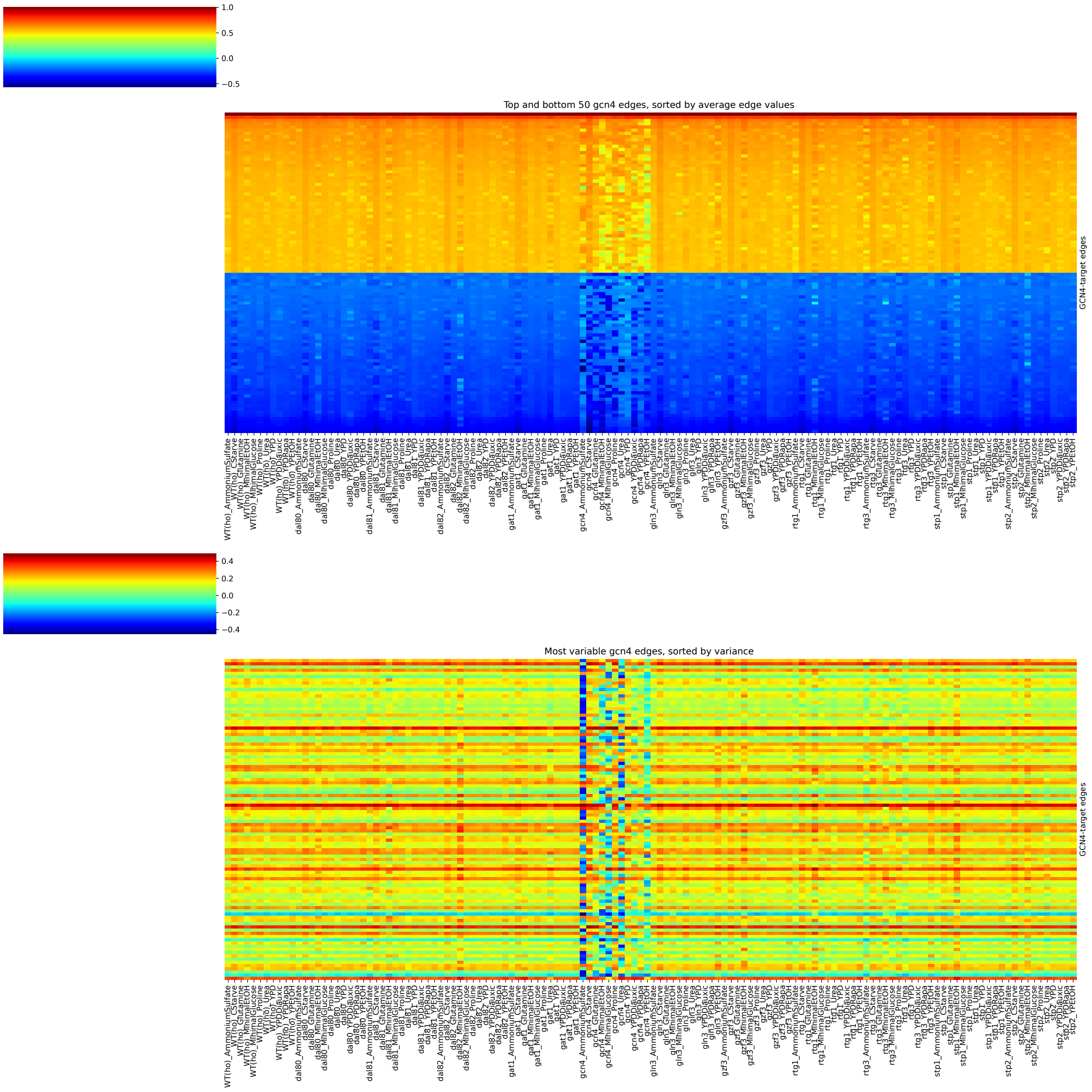

Figure S18: Top: strongest edges between *GCN4* and any other gene. Genes are sorted by the average mean value of the edges (row-wise), and then we select the top and bottom 50 genes. Bottom: most variable edges between *GCN4* and any other gene. Genes are sorted by the average variance value of the edges (row-wise), and then we selected the top 100 genes by variance. In both cases we can see that the effect of *GCN4* knockout (central columns of the heatmaps) produced an effect on the edge weights.

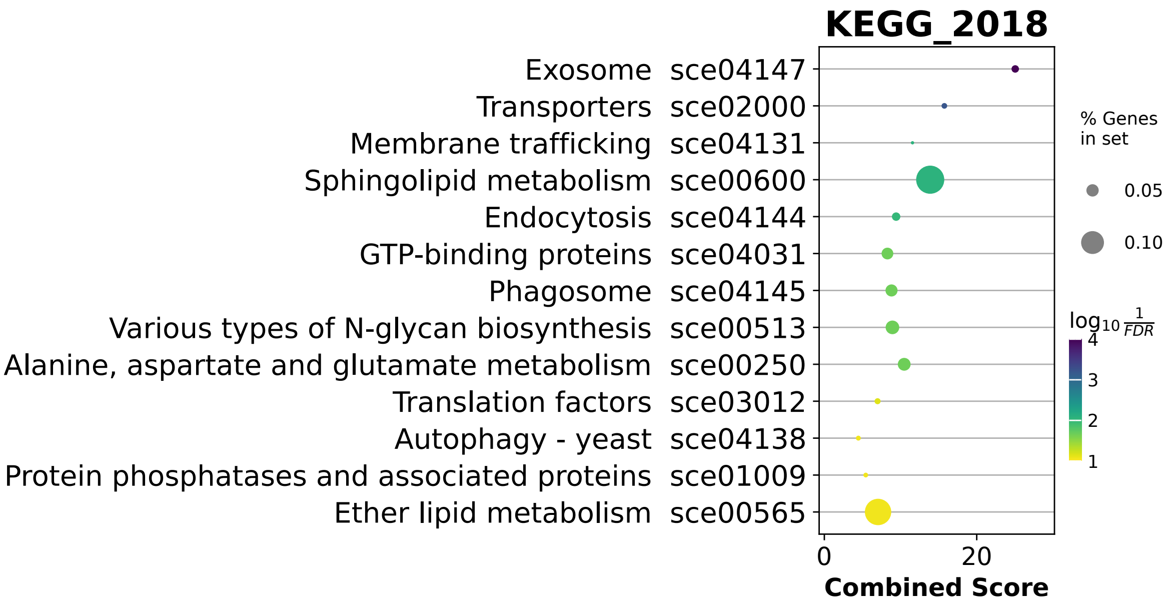

Figure S19: Over-representation analysis of the top 50 genes disrupted by *GCN4* perturbation. The enriched terms belong to the KEGG 2018 pathways.

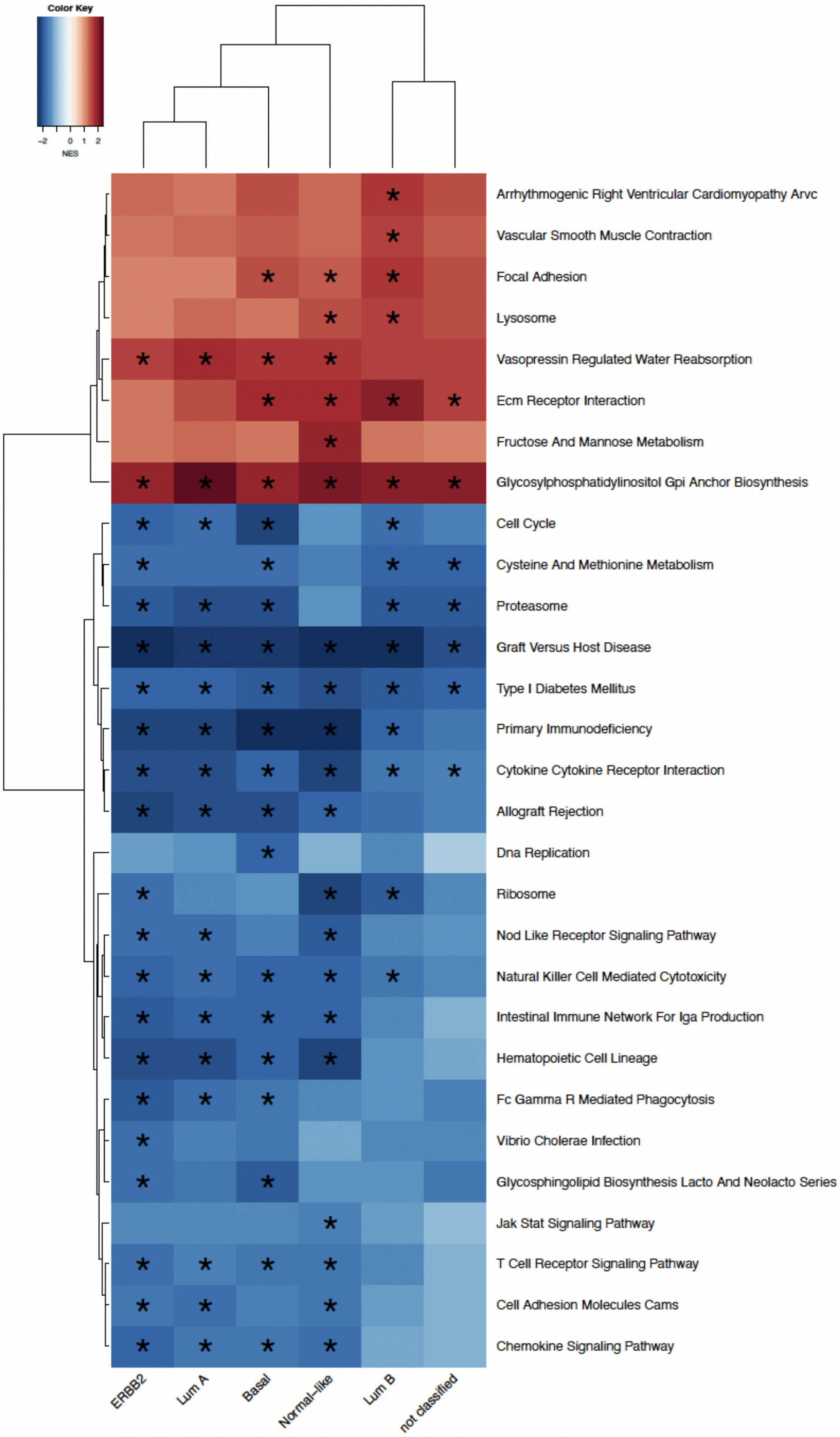

Figure S20: Pathways significantly (at FDR cutoff 0.05) correlated (positively or negatively) with miRNA expression in various breast cancer subtypes: Rows represent pathways and columns represent breast cancer subtypes. Heatmaps are colored by normalized enrichment scores (NES); Pathways positively correlated with miRNA expression are colored red and pathways negatively correlated with miRNA expression are colored blue.

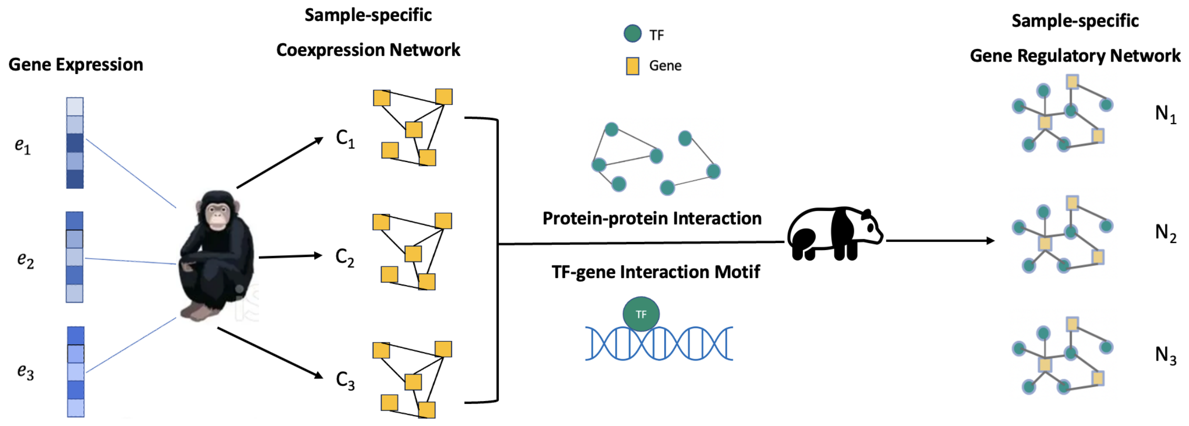

Figure S21: Schematic diagram of the BONOBO-PANDA pipeline: sample-specific co-expression matrices derived by BONOBO are used as input in the PANDA algorithm to derive sample-specific gene regulatory networks.

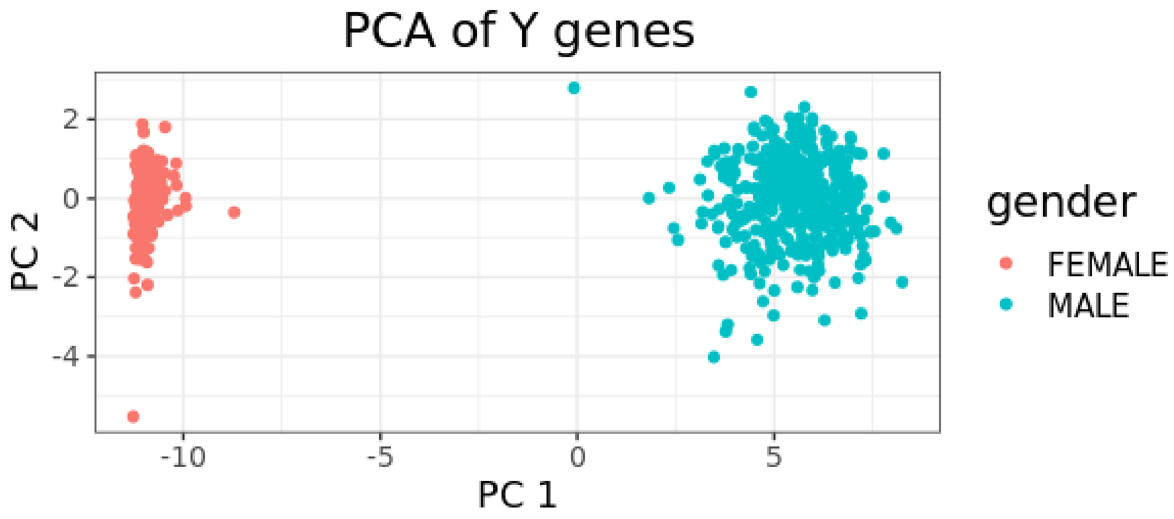

Figure S22: PCA of the log-transformed TPM normalized Y gene expression from GTEx thyroid samples: Self-reported gender matches with chromosomal sex alignment in GTEx thyroid samples.

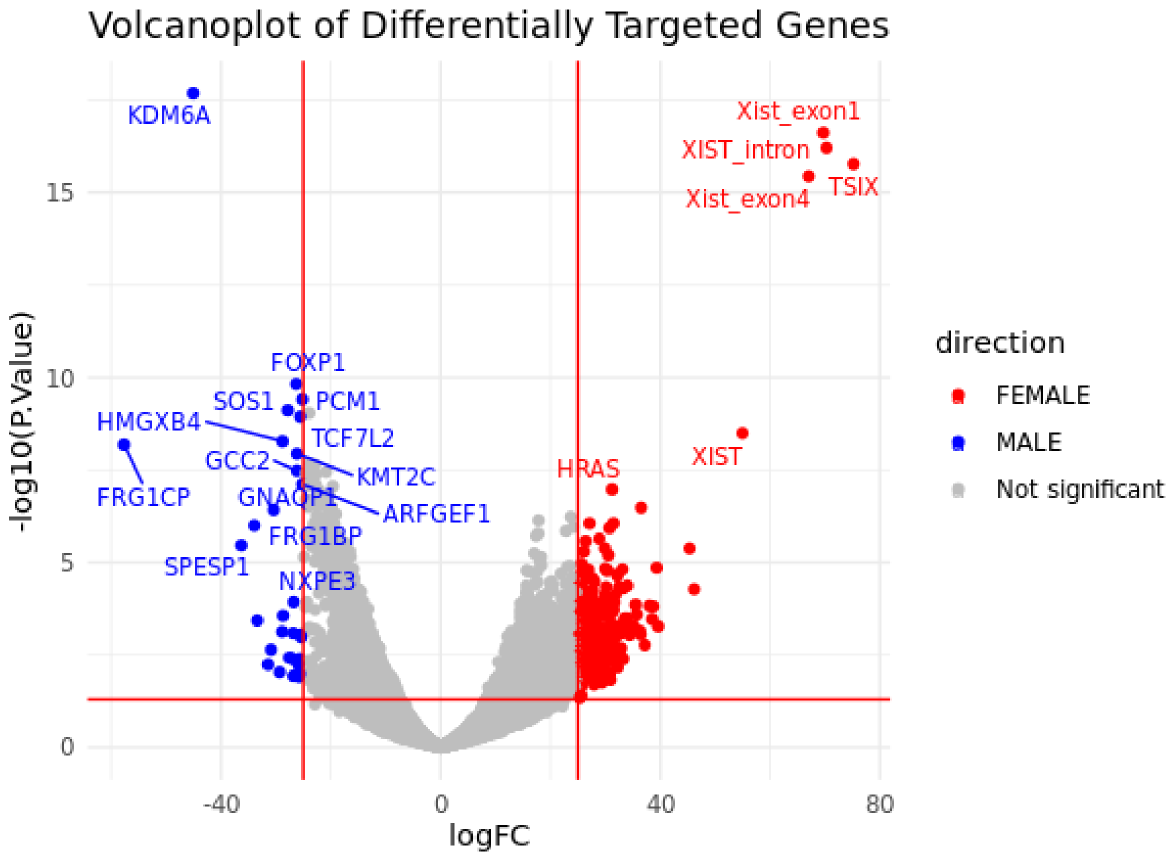

Figure S23: Genes most differentially regulated in males and females in GTEx thyroid samples: volcano plot from “limma” analysis. Genes highly targeted in females compared to males are marked red and genes highly targeted in males compared to females are marked blue.

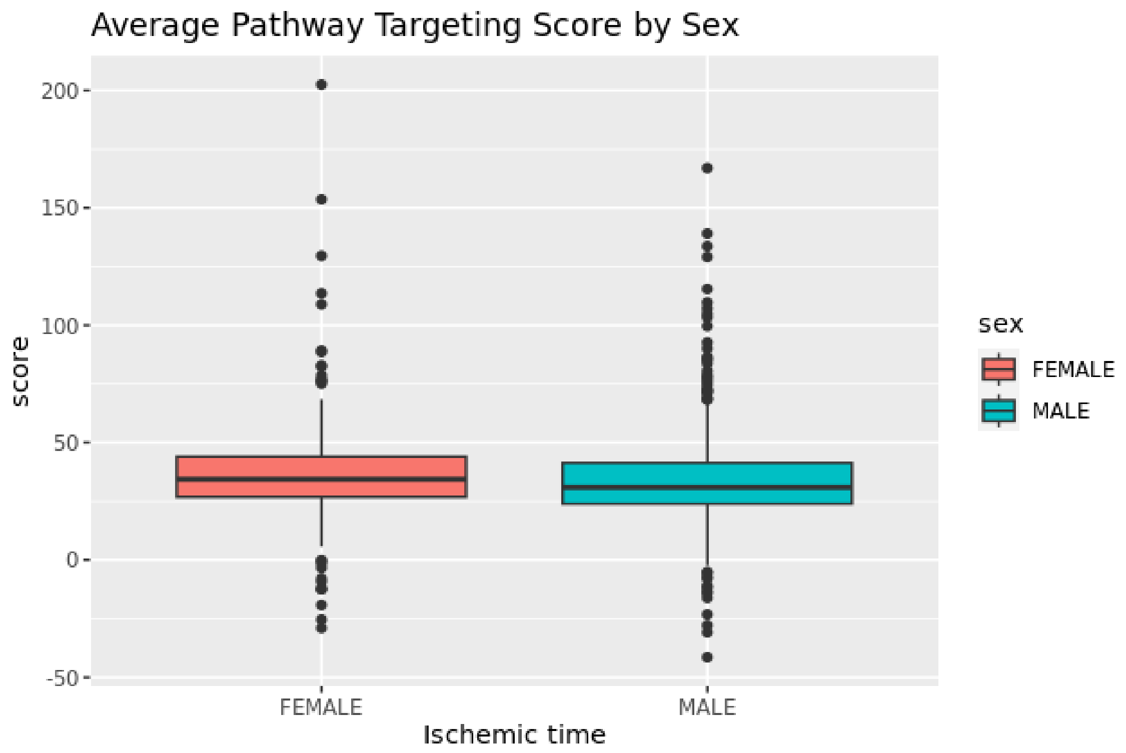

Figure S24: Sex-specific boxplots of average pathway targeting scores computed from individual-specific BONOBO networks. Average pathway targeting scores are computed by averaging over all edges connecting TFs to all genes belonging to the 12 immune-related sex-biased pathways for which individual-specific networks and population-level sex-specific networks give conflicting evidence in favor of sex bias. Females have a higher median targeting score than males, whereas males have more upper-tailed outliers with high individual targeting scores. Individual-specific network analysis suggests that these immune pathways are targeted more in females compared to males. In contrast, group-specific network analysis suggests that these pathways are targeted more in males compared to females.

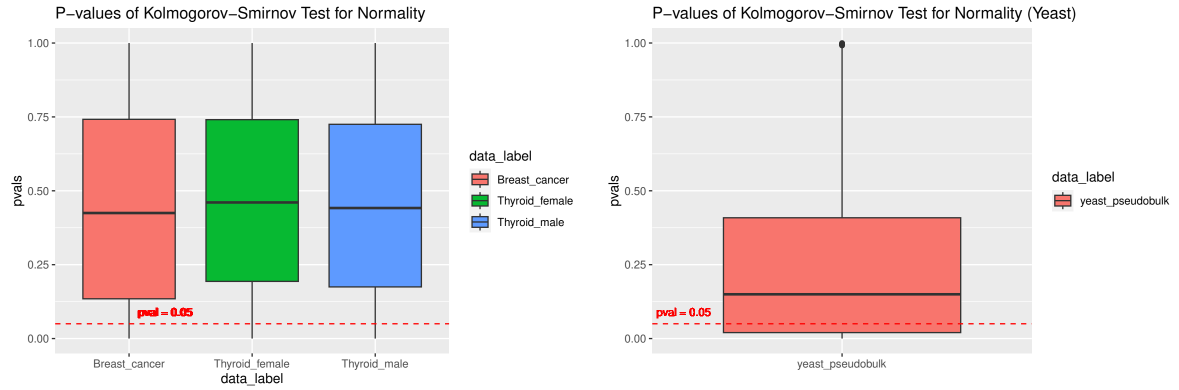

Figure S25: Verifying Gaussian distributional assumption on log-transformed transcriptomics data: (left) boxplot of p-values obtained from Kolmogorov-Smirnov test for testing the null hypothesis whether log-transformed gene expression (from breast cancer subtypes and GTEx thyroid) or miRNA expression (from breast cancer subtypes) data from humans follows a Gaussian distribution; (right) boxplot of p-values obtained from Kolmogorov-Smirnov test for testing the null hypothesis whether log-transformed pseudo-bulked single-cell RNA-sequencing gene expression data from yeast TF knockout experiments follows a Gaussian distribution. In this case we were unable to reject the Gaussian distribution null hypothesis for only 66.61% of genes.

S2 Supplementary Tables

Table S2.1: Mean squared error for BONOBO, LIONESS::Pearson, SPCC, and SWEET on simulated data for varying numbers of genes. In parentheses, we report the MSE scaled by the variance (last column) of the correlations in the true correlation matrix.

Number of Genes	BONOBO	LIONESS	SPCC	SWEET	Variance
100	0.0152<br>(0.0939)	0.1559<br>(0.9635)	0.1739<br>(1.0748)	0.0195<br>(0.1205)	0.1618
250	0.0166<br>(0.1140)	0.1439<br>(0.9883)	0.1550<br>(1.0646)	0.0210<br>(0.1442)	0.1456
500	0.0168<br>(0.1663)	0.1077<br>(1.0663)	0.1126<br>(1.1149)	0.0222<br>(0.2198)	0.1010
750	0.0175<br>(0.2258)	0.0881<br>(1.1368)	0.0910<br>(1.1742)	0.0234<br>(0.3019)	0.0775
1000	0.0176<br>(0.2573)	0.0810<br>(1.1842)	0.0833<br>(1.2178)	0.0238<br>(0.3480)	0.0684
1500	0.0178<br>(0.3002)	0.0738<br>(1.2445)	0.0756<br>(1.2749)	0.0242<br>(0.4081)	0.0593

Pathway Names	NES (individual network)	NES (population network
Activation Of Immune Response	1.83	-1.782
Adaptive Immune Response Based on Somatic Recombination of Immune Receptors Built from Immunoglobulin Superfamily Domains	1.971	-1.496
Antigen Receptor Mediated Signaling Pathway	2.366	-1.699
B Cell Activation	1.73	-1.595
Immune Response Regulating Cell Surface Receptor Signaling Pathway	1.845	-1.972
Immune Response Regulating Signaling Pathway	1.428	-2.314
Interferon Gamma Production	1.482	-1.768
Leukocyte Mediated Immunity	1.854	-1.601
Lymphocyte Mediated Immunity	1.988	-1.697
Positive Regulation of Cell Activation	1.749	-1.736
Production Of Molecular Mediator of Immune Response	2.127	-1.407
Regulation Of Lymphocyte Activation	1.505	-1.933

Table S2.2: Normalized enrichment scores derived from gene set enrichment analysis are shown for 12 immune-related sex-biased pathways for which individual-specific networks (second column) and population-level sex-specific networks (third column) give conflicting evidence in favor of sex bias. Positive NES means the corresponding pathway is targeted by TFs more in females than males, and negative NES means the corresponding pathway is targeted by TFs more in males than females.

References

[1] Miriam Ragle Aure et al. “Identifying in-trans process associated genes in breast cancer by integrated analysis of copy number and expression data”. In: *PloS one* 8.1 (2013), e53014.

[2] Marouen Ben Guebila et al. “The Network Zoo: a multilingual package for the inference and analysis of gene regulatory networks”. In: *Genome Biology* 24.1 (2023), p. 45.

[3] Yoav Benjamini and Yosef Hochberg. “Controlling the false discovery rate: a practical and powerful approach to multiple testing”. In: *Journal of the Royal statistical society: series B (Methodological)* 57.1 (1995), pp. 289–300.

[4] Leonardo Collado-Torres, Abhinav Nellore, and Andrew E Jaffe. “recount workflow: Accessing over 70,000 human RNA-seq samples with Bioconductor”. In: *F1000Research* 6 (2017).

[5] Espen Enerly et al. “miRNA-mRNA integrated analysis reveals roles for miRNAs in primary breast tumors”. In: *PloS one* 6.2 (2011), e16915.

[6] Zhuoqing Fang, Xinyuan Liu, and Gary Peltz. “GSEApy: a comprehensive package for performing gene set enrichment analysis in Python”. In: *Bioinformatics* 39.1 (Nov. 2022), btac757. ISSN: 1367-4811. DOI: 10.1093/bioinformatics/btac757. eprint: https://academic.oup.com/bioinformatics/article-pdf/39/1/btac757/48448971/btac757.pdf. URL: https://doi.org/10.1093/bioinformatics/btac757.

[7] Charles E Grant, Timothy L Bailey, and William Stafford Noble. “FIMO: scanning for occurrences of a given motif”. In: *Bioinformatics* 27.7 (2011), pp. 1017–1018.

[8] Vilde Haakensen et al. “Serum N-glycan analysis in breast cancer patients–relation to tumour biology and clinical outcome”. In: *Molecular oncology* 10.1 (2016), pp. 59–72.

[9] Christopher A Jackson et al. “Gene Regulatory Network Reconstruction Using Single-Cell RNA Sequencing of Barcoded Genotypes in Diverse Environments”. In: *eLife* 9 (2020). Ed. by Naama Barkai and Detlef Weigel, e51254. ISSN: 2050-084X. DOI: 10.7554/eLife.51254. URL: https://doi.org/10.7554/eLife.51254 (visited on 03/08/2023).

[10] Minoru Kanehisa. “The KEGG database”. In: *‘In silico’simulation of biological processes: Novartis Foundation Symposium 247*. Vol. 247. Wiley Online Library. 2002, pp. 91–103.

[11] Gennady Korotkevich et al. “Fast gene set enrichment analysis”. In: *BioRxiv* (2016), p. 060012.

[12] Marieke Lydia Kuijjer et al. “Estimating sample-specific regulatory networks”. In: *Iscience* 14 (2019), pp. 226–240.

[13] John Lonsdale et al. “The genotype-tissue expression (GTEx) project”. In: *Nature genetics* 45.6 (2013), pp. 580–585.

[14] Tata Pramila et al. “The Forkhead Transcription Factor Hcm1 Regulates Chromosome Segregation Genes and Fills the S-phase Gap in the Transcriptional Circuitry of the Cell Cycle”. In: *Genes & Development* 20.16 (Aug. 15, 2006), pp. 2266–2278. ISSN: 0890-9369, 1549-5477. DOI: 10.1101/gad.1450606. pmid: 16912276. URL: http://genesdev.cshlp.org/content/20/16/2266 (visited on 01/20/2022).

[15] Matthew E Ritchie et al. “limma powers differential expression analyses for RNA-sequencing and microarray studies”. In: *Nucleic acids research* 43.7 (2015), e47–e47.

[16] Enakshi Saha et al. “Aging-associated Alterations in the Gene Regulatory Network Landscape Associate with Risk, Prognosis and Response to Therapy in Lung Adenocarcinoma”. In: *bioRxiv* (2024), pp. 2024–07.

[17] Enakshi Saha et al. “Gene regulatory Networks Reveal Sex Difference in Lung Adenocarcinoma”. In: *bioRxiv* (2023). DOI: 10.1101/2023.09.22.559001.

[18] Damian Szklarczyk et al. “STRING v10: protein–protein interaction networks, integrated over the tree of life”. In: *Nucleic acids research* 43.D1 (2015), pp. D447–D452.

[19] Damian Szklarczyk et al. “The STRING database in 2021: customizable protein–protein networks, and functional characterization of user-uploaded gene/measurement sets”. In: *Nucleic acids research* 49.D1 (2021), pp. D605–D612.

[20] Matthew T Weirauch et al. “Determination and inference of eukaryotic transcription factor sequence specificity”. In: *Cell* 158.6 (2014), pp. 1431–1443.

[21] Christopher Wilks et al. “recount3: summaries and queries for large-scale RNA-seq expression and splicing”. In: *Genome biology* 22.1 (2021), pp. 1–40.
